# Cost-benefit potential of sperm function testing to the treatment pathway for unexplained infertility: a modelling study from the UK NHS perspective

**DOI:** 10.1186/s13561-026-00749-5

**Published:** 2026-03-06

**Authors:** Shen Chuen Khaw, Jonathan SY Lee, Huang Lin Khaw, Christopher LR Barratt, Sarah Martins da Silva

**Affiliations:** 1 Reproductive Medicine Research Group, School of Medicine, University of Dundee, Dundee, DD1 9SY UK; 2 Ninewells Hospital and Medical School, Dundee, DD1 9SY UK; 3 Arceptive Limited, Glasgow, G3 8EP UK; 4 Assisted Conception Unit, Ninewells Hospital, Dundee, DD2 1SG UK

**Keywords:** Sperm, Fertilisation failure, Economic evaluation, In vitro fertilisation (IVF), Medically assisted reproduction (MAR)

## Abstract

**Background:**

One-third of infertile couples struggle to conceive despite normal examination and tests. For men, semen analysis is the only widely-adopted diagnostic tool to assess male infertility. However, it is unable to detect abnormal sperm function or accurately predict pregnancy except in cases of complete absence of sperm. Sperm function tests (SFT) could help by directing treatment to intracytoplasmic sperm injection where indicated, improving treatment efficiency and reducing overall cost. However, SFT are not currently routinely used in clinical practice.

**Methods:**

We developed a classification and regression tree model to compare current and alternative fertility treatment pathways from the UK NHS payer perspective over a short-term time horizon covering up to three funded IVF or ICSI cycles. A one-way sensitivity analysis was then performed to evaluate the model’s robustness.

**Results:**

Our model shows that SFT could reduce unnecessary treatment cycles and lower average treatment costs. The base-case model using CatFlux test was associated with a small cost increase at a lower assumed prevalence rate (£9.31 per couple) and cost savings at a higher prevalence rate (£26.65 per couple). One-way sensitivity analysis showed that results were robust to plausible variation in most parameters, with incremental costs remaining close to cost neutrality across scenarios. The model was most sensitive to the probability of pregnancy following ICSI while variation in IVF and ICSI costs and fertilisation failure probabilities had limited impact on results. Based on 18,000 fresh IVF cycles in 2023 alone, this corresponds to a financial impact for the UK NHS ranging from an additional cost of £167,500 to a cost saving of £479,700.

Beyond economic benefits, reliable SFT would improve overall treatment success by reducing IVF affected by fertilisation failure, enabling targeted selection of medically assisted reproduction (MAR) and faster time to pregnancy. It spares countless couples the emotional distress, financial burden, and health risks associated with repeated fertility treatments.

**Conclusion:**

Based on a clinical pathway model, integrating SFT into fertility treatment pathways has the potential to generate savings for healthcare systems. More importantly, it enables personalised and precise MAR, reducing the risks and inefficiencies of trial-and-error treatment cycles as well as improving patient outcomes.

**Supplementary Information:**

The online version contains supplementary material available at 10.1186/s13561-026-00749-5.

## Introduction

One in six couples experience infertility globally [1] and in as many as a third of these cases, the cause is unclear [2, 3]. These couples struggle to conceive despite having normal semen analysis results in the male partner and no apparent fertility issues in the female partner. This condition is known as unexplained infertility which leaves many couples desperate when their test results come back normal, yet they are not able to conceive [3]. In these cases, there is no evidence base for any treatment except for in vitro fertilisation (IVF). However, couples may still resort to using unproven medications and unsubstantiated supplements, which may be ineffective and harmful in some cases [4, 5]. Additionally, the pursuit of unnecessary treatments can lead to physical risks, financial burdens and emotional strain 6]. This can negatively impact overall quality of life and may even lead to strain in personal relationships [7].

In the United Kingdom, stringent NHS criteria may delay access to fertility treatment [8]. Generally, couples with infertility must try to conceive for at least 12 months before being referred to specialist care. When routine evaluations such as semen analysis, ovulation assessment and tubal patency testing reveal no abnormalities, couples with unexplained infertility are advised to continue trying naturally for up to 24 months, owing to the higher likelihood of natural pregnancy compared with in vitro fertilisation (IVF). After being seen by a fertility specialist, further investigations and trial-and-error treatment cycles would extend this timeline further, resulting in couples only receiving medically assisted reproduction (MAR) years after first attempting to conceive. By then, their reproductive and personal circumstances may have changed, potentially leading to a poorer prognosis, such as due to age-related decline in oocyte quality. Moreover, extended attempts at natural conception are unlikely to succeed in men with sperm dysfunction. Therefore, the incorporation of an accurate sperm function test (SFT) could enable early diagnoses, streamline clinical management and facilitate personalised care for these couples.

Traditional semen analysis, although universally used in reproductive care, provides limited predictive value for successful fertilisation. Semen analysis primarily assesses basic sperm characteristics without evaluating crucial functional aspects [9, 10]. This lack of precise diagnostic methods is contributed by a broader issue: male infertility is frequently overlooked in healthcare, with inadequate recognition of its implications for systemic health and reproduction [11, 12]. Fertility care is typically provided within gynaecology departments, staffed by fertility specialists who are trained in women’s health. Consequently, male partners are often relegated to a generic workup despite accounting for nearly half of all infertility cases. This approach bypasses underlying causes without targeted interventions, which leads to missed opportunities for targeted treatment and a disproportionate burden on female partners [11].

Fertilisation failure affects approximately 5–10% of IVF cases [13, 14]. If sperm function testing could identify cases where fertilisation failure is likely to occur, it could help couples make informed choices and guide clinical treatment decisions. For example, an early recourse to intracytoplasmic sperm injection (ICSI) could be recommended in the context of abnormal sperm function and high risk of fertilisation failure during IVF. Despite the potential benefits of SFT, no such test is currently available in routine clinical practice and research into sperm function testing remains a low priority [15].

Over the last 60 years, various sperm function tests have been developed to evaluate sperm quality, including assessments of sperm-cervical mucus penetration, zona pellucida binding, DNA fragmentation, oxidative stress, hyaluronan binding, and acrosome reaction. However, these tests have proven unreliable in predicting fertilisation success [16, 17] and require advanced laboratory techniques [18] which are impractical for clinical settings. However, the recent development of the CatFlux test, a diagnostic test that measures activity in the sperm-specific calcium channel (CatSper), required for fertilisation, shows promise for predicting fertilisation failure [19, 20]. The CatFlux test [20] is commercially available (www.truion.de) and has also been introduced in several European countries but it has yet to be evaluated and adopted by the UK’s NHS.

Unlike prior economic evaluations that focus on comparing IVF and ICSI as treatment strategies [21, 22], this study evaluates the economic consequences of introducing a diagnostic intervention earlier in the fertility care pathway. Using a decision-analytic model from the UK NHS perspective, we estimate how SFT could alter downstream treatment allocation, resource use and costs. This work contributes to the health economics literature by understanding whether improving diagnostic precision in male infertility can reduce inefficient use of assisted reproductive technologies.

## Materials and methods

Two authors (SCK and HLK) conducted a literature search using keywords such as “unexplained infertility”, “in vitro fertilisation”, “IVF”, “intracytoplasmic sperm injection”, “ICSI”, “cost,” “fees,” and “fertilisation failure” across primary databases, including PubMed, EMBASE, and MEDLINE. This search focused on peer-reviewed English publications from their inception to December 2025. Additionally, publicly available websites were examined to gather relevant information on the costs associated with tests and treatments. The authors screened the titles and abstracts for potentially eligible studies and articles were reviewed for inclusion with SMdS. Conflicts were resolved through consensus or by discussion with a senior author. Ethical approval was not required as only published data were used.

### Clinical pathway analysis

We extracted data from five NHS trusts across the United Kingdom, revealing that semen analysis costs between £100 and £150, whereas IVF costs approximately £5000 per cycle, with a further £1000 for ICSI [23–27].

**Fig. 1:**
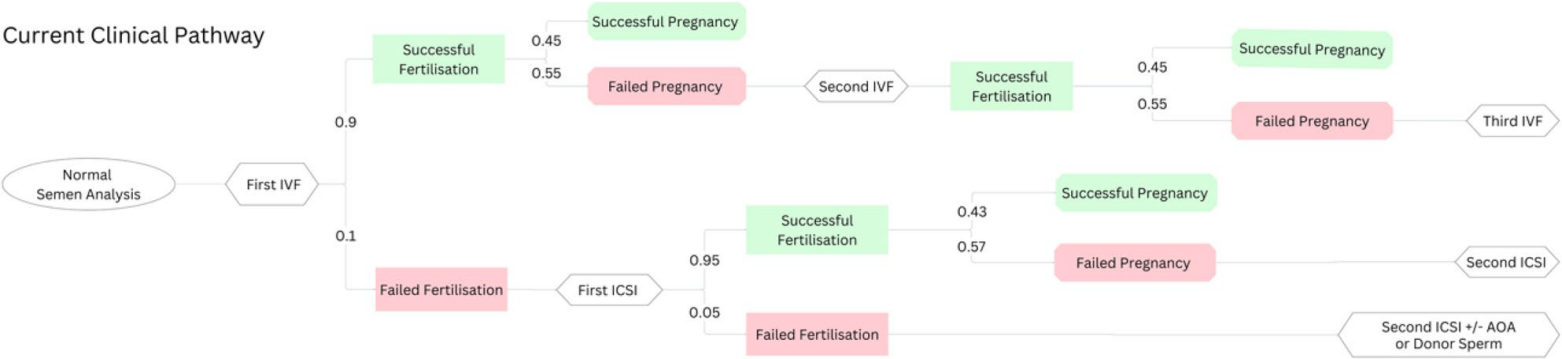
Current clinical pathway of unexplained infertility with outcomes and costs associated in the UK. In the current clinical treatment pathway, when a normal semen analysis is reported, couples with unexplained infertility are typically recommended for IVF. Treatment outcomes are then categorised based on the probability of successful or failed fertilisation, followed by either a successful or failed clinical pregnancy. For couples experiencing failed fertilisation, the next cycle should be ICSI. Each treatment and its associated outcomes are assigned a cost, reflecting the financial burden at each stage of the process. The total cost is then calculated by combining the overall expenditure based on treatment outcomes. *AOA – Artificial Oocyte Activation*

Our study utilised a decision-tree model, informed by a classification and regression tree (CART) framework [28], to evaluate the economic implications of incorporating a SFT into the current treatment pathway (Fig. 1) for couples with unexplained infertility. The analysis was conducted from the perspective of the UK NHS, with a short-term time horizon reflecting up to three publicly funded cycles of IVF or ICSI, in accordance with NICE guidance [8].

Based on real world clinical decision making, the treatment pathway for couples with unexplained infertility typically starts with IVF. ICSI is then offered for subsequent attempts if low or no fertilisation has occurred. Fig. 1 illustrates standard clinical practice pathways and includes various scenarios and corresponding outcomes. The pathways model the probability of occurrence, the outcomes (fertilisation and pregnancy) and associated costs. The cumulative outcomes along the branches for each strategy contribute to the CART evaluation. In essence, the treatment pathways serve as the foundation for a practical analysis of the financial implications and success rates of current infertility treatment approaches.

**Fig. 2:**
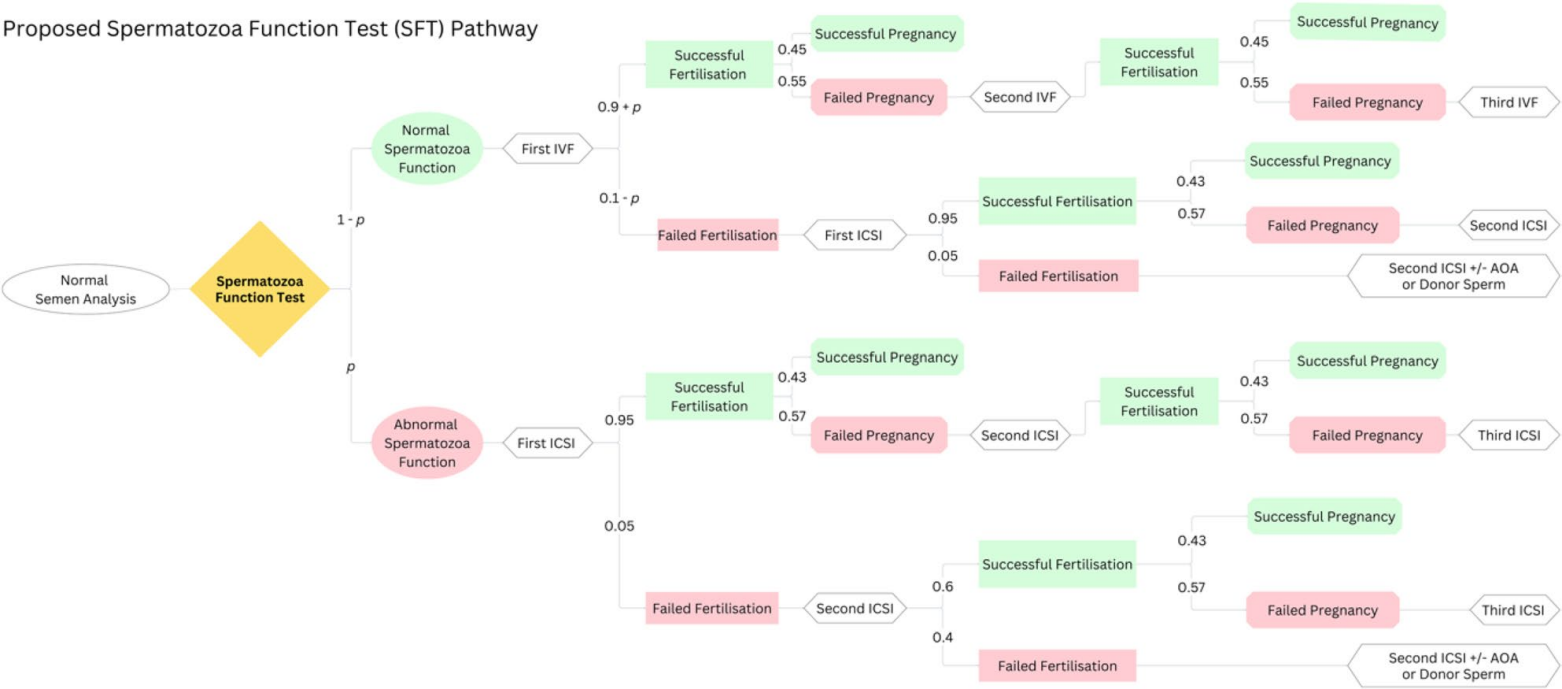
Decision-analytical model comparing the outcomes and costs associated with a proposed alternative SFT pathway. In this model, normal semen analysis is paired with SFT. Couples with normal sperm function are directed towards IVF, with the probability of fertilisation and pregnancy outlined in the pathway. ICSI is subsequently recommended where treatment has been complicated by low or no fertilisation. Conversely, couples identified with abnormal sperm function are offered ICSI for their first cycle of treatment. The costs associated with each step of both treatment pathways are mapped out in the model to assess financial impact. *AOA – Artificial Oocyte Activation*, $p$ – *sensitivity of the sperm function test* (0.01 < *p* < 0.099)

We propose an alternative pathway incorporating an SFT, outlined in Fig. 2. This alternative model directs men with a normal semen analysis, yet abnormal SFT result immediately to ICSI, thus eliminating an initial cycle of IVF complicated by failed fertilisation.

The model also factors in the costs and outcomes for both IVF and ICSI pathways, starting from a key decision node representing the treatment choice. The pathways delineate probabilities, outcomes (fertilisation and pregnancy) and associated costs, forming the basis for a cost-benefit evaluation of infertility treatment strategies. The assumptions underlying this model encompass the expected costs associated with IVF and ICSI (Table 1). Specifically, the likelihood of encountering total fertilisation failure (TFF) during IVF procedures, estimated to range between 5 and 10% [13, 14] and 1–5% in ICSI [6, 29, 30]. While ICSI is typically reported to have a fertilisation failure rate of 1–3% [6], a recent large open-label randomised controlled trial has observed rates as high as 5% [29]. Therefore, this maximum failure rate of 5% has been used to estimate the minimum potential cost savings.

Moreover, the model considers the probability of successful pregnancy after IVF (45%) or ICSI (43%) for unexplained infertility [31]. The model assumes that the likelihood of fertilisation and pregnancy remains constant across successive IVF or ICSI cycles, regardless of whether the SFT is administered. We have used 40% as the maximum rate for recurrent fertilisation failure after ICSI [32] to account for oocyte factors and the highest possible cost incurred after two unsuccessful ICSI cycles to ensure that we calculate the minimum amount of cost savings.

**Table 1:** Model parameters, assumptions and data sources

Parameter (per patient)	Base-case value	Range	Data sources
IVF cost	£5000	£4500 – £6000	[23–27]
IVF fertilisation failure	10%	5% – 10%	[13, 14]
IVF pregnancy success	45%	23% – 45%	[31, 33–35]
ICSI cost	£6000	£5500 – £7000	[23–27]
ICSI fertilisation failure	5%	1% – 5%	[6, 29, 30]
ICSI recurrent fertilisation failure	40%	10% – 40%	[32, 36]
ICSI pregnancy success	43%	16% – 43%	[31, 35]
False positive rate of CatFlux test	1.7%	1.7%	[20]

Comparing the two models, both treatment pathways start with an initial round of IVF, followed by ICSI in cases of fertilisation failure. However, the SFT pathway incorporates SFT sensitivity in detecting fertilisation failure, represented by $p$, ranging from 0.001 to 0.099. In the SFT pathway, the rate of fertilisation failure after IVF is expected to decrease, as only patients with normal SFT results are offered IVF, while those with abnormal results are directed to ICSI. This approach aims to evaluate the change in costs resulting from a decrease in fertilisation failure after initial IVF.

### Cost-benefit analysis

According to initial calculations, the expected cost per patient in the UK following current clinical pathway (Fig. 1) is £9,791.15. These costs encompass cases of failed fertilisation, unsuccessful pregnancies, and up to three IVF or ICSI cycles, in line with UK NHS funding limits. It is expected that improving the sensitivity of the SFT will reduce the overall cost of treatment.

We used the decision tree (Fig. 2) to calculate expected savings and expected costs across different success rates of the SFT. In the SFT pathway, we have further divided the cost associated with patients who are treated with IVF first with those that are streamlined to ICSI, based on the results of their SFT.

Our study adopts an NHS treatment perspective and uses a short-term decision-analytic model covering up to three publicly funded IVF or ICSI cycles which is consistent with NICE guidance [8]. Given the short time horizon, discounting was not applied. The analysis is based on a cost-consequence evaluation, as health outcomes such as live birth and quality-adjusted life years (QALYs) could not be robustly estimated due to limited evidence linking sperm function testing to long-term outcomes. We have discussed this limitation is explicitly below.

### Sensitivity and specificity

The sensitivity of the SFT is the ability for the test to accurately detect sperm samples that will lead to fertilisation failure, expressed on a scale of 0% to 100% [37]. Higher values mean that the SFT is able to accurately identify more samples that may result in fertilisation failure. On the other hand, specificity is the ability for the SFT to correctly produce a negative result in sperm samples that will not result in fertilisation failure, also ranging between 0% and 100% [37]. A higher specificity indicates fewer normal sperm samples are mistakenly classified as abnormal.

### Expected value of the SFT pathway

The expected cost of the IVF pathway is largely influenced by the probability of fertilisation failure in the first IVF cycle when an SFT is used. As the sensitivity of the SFT increases, the failure rate of the initial IVF cycle is expected to decrease. This is because a more sensitive SFT can better identify patients who would benefit from being directed to the ICSI pathway, thereby reducing the incidence of IVF failures.

### Total expected value of the SFT pathway

The expected cost of the SFT pathway can be calculated based on the varying sensitivity of the SFT. The formula for calculating the expected value is:

$$E\left(X\right)={X}_{1}\left(1-p\right)+{X}_{2}\left(p\right)$$

Where:

- *E(X)* represents the expected cost of the SFT pathway
- *X*_*1*_​ is the expected cost of the IVF treatment pathway.
- *X*_*2*_​ is the expected cost of the ICSI treatment pathway.
- *p* is the sensitivity of the SFT.

As the sensitivity (*p*) of the test varies, we can compute the combined expected cost at different intervals, providing us with the results outlined in Table 2. The code and interactive model used for the cost-benefit analysis in the UK and its results are detailed in Appendix A.

### CatFlux test

After developing the hypothetical model, we applied the CatFlux test [20]. The CatFlux was used as an illustrative example as it identifies 1.2% to 2.3% of fertilisation failure in normozoospermic men [19] with a sensitivity of 100% and specificity of 98.3% [20]. However, its regulatory status, cost structure and real-world performance within NHS laboratories remain to be established. Furthermore, as we do not have an available NHS pricing for CatFlux, we have estimated the cost of the CatFlux test to be approximately £50 (EUR55) per test based on available European market commercial pricing to examine their impact on cost savings [38].

While the positive and negative predictive values (PPV and NPV) were not explicitly calculated, we have implicitly analysed their influence through a variation in the assumed prevalence of CatSper dysfunction, test sensitivity and test specificity as these factors will directly influence the proportion of true positives, false positives and downstream treatment outcomes [39, 40].

A one-way sensitivity analysis (OWSA) was conducted to assess the robustness of model results to uncertainty in key parameters [41]. Each parameter was varied independently across a plausible range while holding all other parameters at their base-case values. The parameters explored in the OWSA included IVF and ICSI cycle costs, fertilisation failure rates for IVF and ICSI, probability of recurrent fertilisation failure following ICSI, and pregnancy success rates for IVF and ICSI. Ranges for each parameter were informed by published evidence and clinical plausibility (Table 1). The impact of parameter variation on expected costs was examined to identify key drivers of cost savings and to assess whether the introduction of CatFlux remained cost-saving across plausible scenarios.

## Results

### Proposed SFT model

Applying the concept of expected value and assuming varying predictive values for fertilisation failure in normozoospermic men within the UK Health System, we observed modest cost savings per patient (£3.45) when the SFT sensitivity is 1%. As the test sensitivity increases, cost savings rise correspondingly to a maximum of £290 per patient when the test’s sensitivity reaches 99% (Table 2). However, it should be noted that the net financial benefit will depend on the cost of the SFT, which was not incorporated into the generic SFT analysis.

**Table 2:** The expected cost of the proposed SFT pathway decreases as the sensitivity of the sperm function test (SFT) increases based on United Kingdom (UK) costings

Sensitivity of SFT, *p*	Expected Cost of Current Clinical Pathway (£)	Expected Cost of IVF-first route within SFT pathway*, X1(1-*p*) (£)	Expected Cost of ICSI-first route within SFT pathway*, X2(*p*) (£)	Expected Cost of SFT Pathway*, E(X) (£)	Expected Savings of SFT Pathway* (£)
0	9791.15	9791.15	0	9791.15	0
0.001	9791.15	9776.08	11.62	9787.7	3.45
0.002	9791.15	9761.02	23.25	9784.26	6.89
0.003	9791.15	9745.96	34.87	9780.84	10.31
…	…	…	…	…	…
0.097	9791.15	8378.36	1127.48	9505.84	285.31
0.098	9791.15	8364.31	1139.11	9503.42	287.73
0.099	9791.15	8350.28	1150.73	9501.01	290.14

The expected cost of the SFT pathway, denoted as E(X), is determined by aggregating the expected costs of two distinct routes: the IVF-first route, represented by X₁(1-p), and the ICSI-first route, represented by X₂(p), where *p* denotes the sensitivity associated with the SFT. The expected savings from the SFT pathway are then computed by taking the difference between the expected cost of the current clinical pathway and that of the SFT pathway in the United Kingdom. All costs are reported in GBP (£) and represent expected per-patient costs across up to three funded treatment cycles, before accounting for the unit cost of the SFT. For a comprehensive breakdown of these calculations, please refer to Appendix A

*Costs shown are prior to subtracting the cost of the SFT

### CatFlux test application

**Fig. 3:**
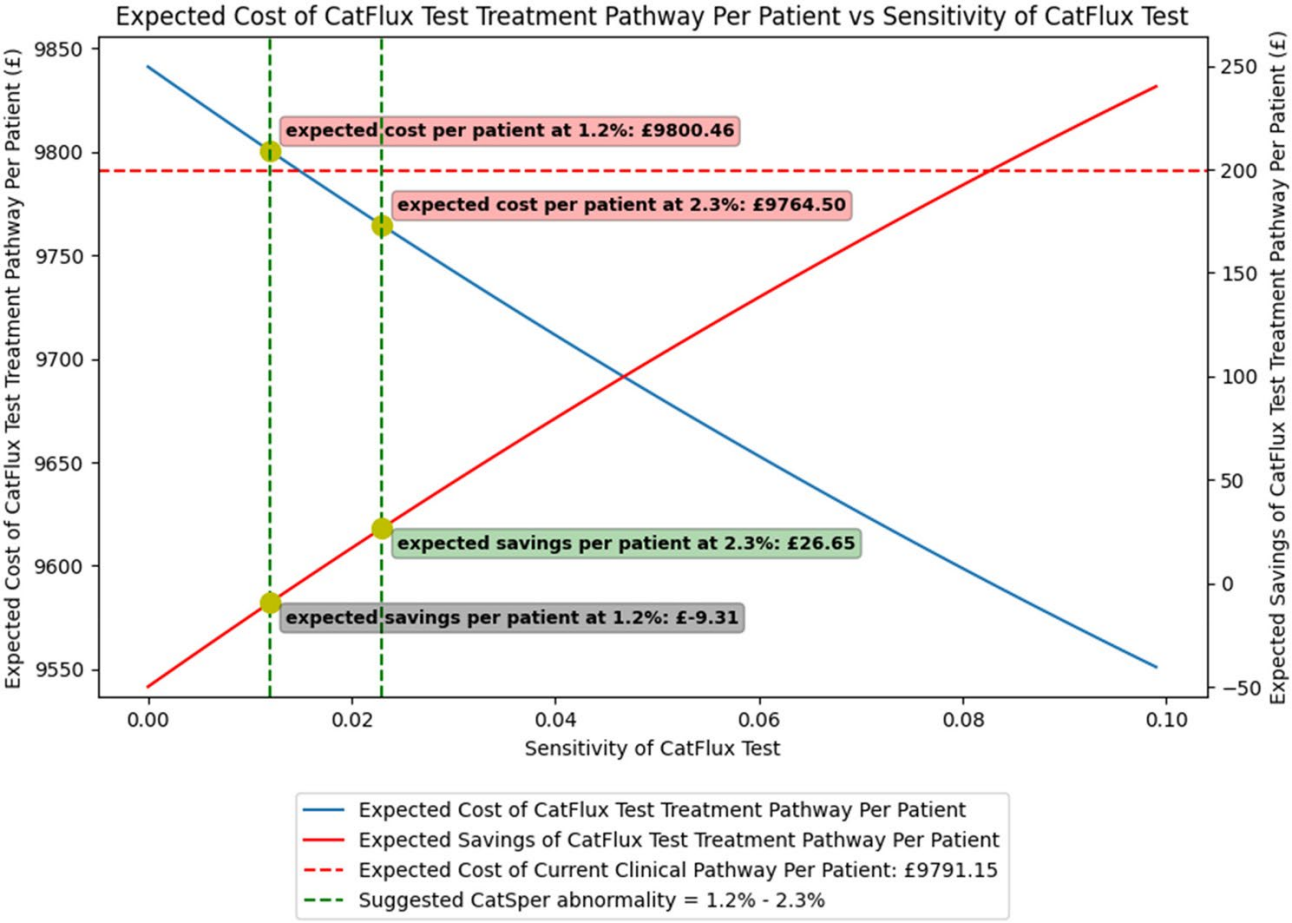
A visual interpretation of expectant treatment cost and resultant savings using the CatFlux Test. The expected cost of the proposed SFT treatment pathway is analysed in relation to the sensitivity of the SFT. The dotted red line illustrates the expected cost of the current clinical pathway per patient in the UK, which includes up to three cycles of IVF or ICSI, depending on treatment outcomes. In contrast, the solid blue line represents the expected cost of the SFT treatment pathway, which also includes up to three cycles of IVF or ICSI but is influenced by the sensitivity of the SFT and subsequent treatment outcomes. The solid red line denotes the anticipated cost savings per patient when using the SFT treatment pathway. As the sensitivity of the SFT increases, the expected cost per patient along the SFT treatment pathway decreases, resulting in greater savings per patient. The dotted green line represents an expected predictive rate of between 1.2% to 2.3% fertilisation failure using the CatFlux test, leading to an incremental cost ranged from an increase of £9.31 to a cost saving of £26.65 per couple

In the base-case analysis, the cost savings following the introduction of the CatFlux test was dependent on the prevalence of CatSper abnormality. At a predictive rate of 1.2% – 2.3%, the mean expected cost per couple was between £9764.50 – £9800.46 in the CatFlux pathway compared with £9791.15 in the standard care pathway, corresponding to an expected cost saving of between -£9.31 to £26.65 per couple (Fig. 3).

The cost difference was achieved without modelling differences in overall pregnancy rates between strategies, reflecting the assumption that CatFlux affects treatment allocation rather than biological treatment effectiveness. Any cost savings arose from more efficient initial pathway selection, leading to fewer cycles characterised by fertilisation failure and a reduction in downstream treatment escalation. However, at lower assumed prevalence of sperm dysfunction, the effective positive predictive value of the SFT would be reduced, increasing the proportion of false-positive results and attenuating cost savings, whereas higher prevalence improves predictive value and enhances economic benefit.

### One-way sensitivity analysis

The results of the one-way sensitivity analysis are presented as expected cost savings per couple associated with the CatFlux pathway compared with standard care, evaluated at two alternative values of the prevalence rate (1.2% and 2.3%). All analyses are outlined in Table 3 and include the cost of the CatFlux test (£50 per test).

In the base-case scenario, the CatFlux pathway resulted in a small cost increase at a prevalence rate of 1.2% (£9.31 per couple) but became cost saving at a higher prevalence rate of 2.3%, with savings of £26.65 per couple. The cost difference indicates a threshold effect whereby the economic value of CatFlux depends on the prevalence of abnormal sperm function and the proportion of couples appropriately reallocated to first-line ICSI.

Reducing the unit cost of IVF to £4,500 decreased the magnitude of cost savings, resulting in a cost increase of £14.86 per couple at a 1.2% prevalence rate and savings of £15.90 at 2.3%. Conversely, increasing the IVF cost to £6,000 strengthened the economic case for CatFlux, producing savings of £1.79 at 1.2% and £48.14 at 2.3%. Lowering the IVF fertilisation failure probability to 5% similarly reduced savings, yielding a cost increase of £12.48 per couple at 1.2% and savings of £20.57 at 2.3%. Contrarily, a lower IVF pregnancy success of 23% increased the value of the CatFlux pathway, with savings of £22.13 at a 2.3% prevalence rate, although a small cost increase (£12.01) remained at 1.2%. These show that the SFT is more economically beneficial in systems where IVF is more expensive or less efficient.

When the cost of ICSI was reduced to £5,500, there was a cost increase of £8.08 per couple at 1.2% and savings of £29.22 at 2.3% when compared to the current clinical pathway. Increasing the cost of ICSI to £7,000 did not improve savings at the lower prevalence rate (-£11.78) but maintained positive savings of £21.51 at 2.3%. Reducing the ICSI fertilisation failure probability to 1% modestly attenuated savings, with a cost increase of £8.22 at 1.2% and savings of £28.77 at 2.3%. Similarly, reducing the probability of recurrent fertilisation failure after ICSI to 10% resulted in savings of £27.54 at 2.3%, while remaining cost increasing at 1.2% (£8.85). When ICSI pregnancy success was reduced to 16%, the CatFlux strategy resulted in cost increases at both prevalence rates, with losses of £34.31 at 1.2% and £21.65 at 2.3%. These findings suggest that the SFT remains economically attractive under plausible improvements in ICSI outcomes and a lower unit cost, while poor pregnancy outcomes would make it inefficient.

**Table 3:** One-way sensitivity analysis utilising CatFlux test

Parameter varied	1.2% Predictive Rate (£)	2.3% Predictive Rate (£)
Base case (control)	-9.31	26.65
IVF cost ↓ £4,500	-14.86	15.90
IVF cost ↑ £6,000	1.79	48.14
IVF fertilisation failure ↓ 5%	-12.48	20.57
IVF pregnancy success ↓ 23%	-12.01	22.13
ICSI cost ↓ £5,500	-8.08	29.22
ICSI cost ↑ £7,000	-11.78	21.51
ICSI fertilisation failure ↓ 1%	-8.22	28.77
ICSI recurrent failure ↓ 10%	-8.85	27.54
ICSI pregnancy success ↓ 16%	-34.31	-21.65

Negative values indicate higher cost vs. standard care. The one-way sensitivity analysis includes £50 CatFlux test cost

**Fig. 4:**
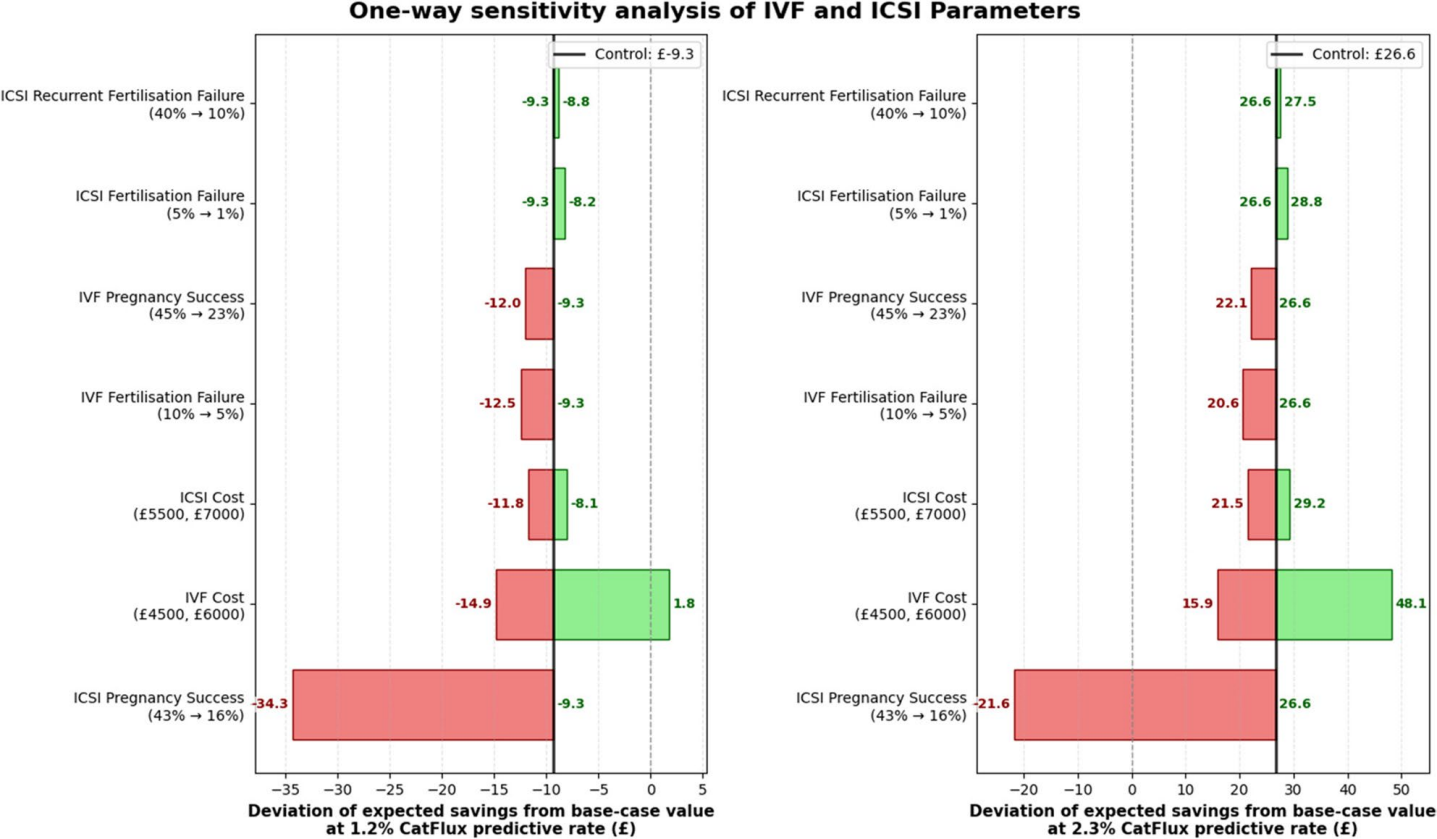
One-way sensitivity analysis of the CatFlux test pathway. This figure illustrates a tornado-plot visualisation of the one-way sensitivity analysis which shows the impact of varying individual IVF and ICSI pathway parameters on cost saving per couple relative to the current clinical pathway. Results are shown for the two assumed CatFlux predictive rates of 1.2% (left panel) and 2.3% (right panel). The horizontal bars represent the deviation in expected cost savings from the base-case value when each denoted parameter is varied, while all other parameters are held constant. The vertical reference line denotes the base-case cost saving. Parameters are ordered by magnitude of impact, with longer bars in either direction indicating greater deviation in results. The analysis demonstrates that uncertainty is driven primarily by the probability of pregnancy following ICSI, with other cost and fertilisation parameters having a comparatively limited effect on overall costs

Overall, the OWSA demonstrated that the cost impact of CatFlux is sensitive to assumptions regarding treatment effectiveness and the proportion of couples reallocated by the test. Cost savings were consistently observed at a prevalence rate of 2.3% across most parameter variations, whereas results at 1.2% were more sensitive and frequently cost increasing. The analysis indicates that the CatFlux pathway is most likely to be cost saving in settings characterised by higher IVF costs, higher IVF failure rates and moderate to high effectiveness of ICSI, while its economic value diminishes when ICSI pregnancy success is substantially reduced. These findings have been illustrated in Fig. 4.

### UK-wide implications

In the United Kingdom alone, approximately 18,000 fresh IVF cycles procedures (without ICSI) were conducted in 2023 [42]. Considering this, annual cost savings for UK fertility services using the proposed SFT pathway could range from about £62,000 at 1% test sensitivity to nearly £5.2 million at 99% sensitivity, prior to subtracting the modest cost of the SFT. Employing the CatFlux test as a hypothetical SFT could potentially lead to an increased cost of £167,500 or yield savings of up to £479,700 annually depending on test performance and NHS uptake.

## Discussion

Our CART Analysis, based on real-world clinical decision-making pathway in the UK NHS, and an alternative pre-treatment diagnostic approach, indicates that incorporating SFT could result in substantial financial savings. This holds true even in the worst-case scenario, where fertility treatment outcomes remain poor despite ICSI.

Moreover, the economic implications of implementing a specific and sensitive SFT go beyond mere monetary savings, playing a major role in fostering increased efficiency within a fertility centre and providing effective treatment strategies. From a commissioning perspective, SFT represents a diagnostic investment with potential to reallocate resources away from ineffective IVF cycles toward more appropriate use of ICSI, thereby improving throughput within capacity-constrained NHS fertility resources, including laboratory capacity, clinical time and publicly funded treatment slots. Given the already high demand and workload for specialist doctors and laboratory staff required in assisted reproduction [43], these efficiencies could be particularly valuable in mitigating growing waiting times. Reducing avoidable IVF failure may therefore generate opportunity costs savings, enabling reallocation of resources to other patients or services. It would also be highly beneficial to provide earlier treatment as initiating care at a younger age, especially in female partners, would lead to better reproductive outcomes [44].

Our vision is that access to SFT would allow male partners to receive an early and accurate diagnosis, allowing couples to receive timely and personalised treatment. The test could potentially be integrated into routine fertility assessments in couples presenting with infertility. Despite normozoospermia, if sperm dysfunction is detected, men could be appropriately directed to personalised fertility treatment rather than unnecessarily enduring the standard two-year period of attempting natural conception or trying intrauterine insemination as fertilisation is unlikely to be successful in these cases. Early identification of sperm dysfunction streamlines fertility consultations and allows clinicians to recommend the most effective treatment, rather than relying on empirical trials of IVF.

Moreover, accurately identifying couples who would benefit from ICSI can help minimise the need for multiple ‘trial-and-error’ IVF cycles, reducing the physical suffering and emotional burden from multiple treatment cycles [45, 46]. Mild ovarian hyperstimulation syndrome (OHSS) which occurs in 1 in 3 cycles and moderate or severe OHSS in approximately 1–2% of IVF or ICSI cycles, are examples of such risks [47, 48]. Additionally, women must self-inject medications, often experiencing side effects and hormonal imbalance as a result [49]. Therefore, being able to offer effective care plans not only reduce treatment-related risks, but also mitigates inconveniences, emotional strain and the potential relationship breakdowns that often accompany failed cycles [49, 50].

In this study, we used the CatFlux test to model costs as it is a commercially available SFT [20, 38]. Based on the CART analysis, the estimated cost impact ranges from an incremental cost of £9.31 to a cost saving of £26.65 per couple. The one-way sensitivity analysis demonstrates that the economic findings are generally robust to differences in key cost and reported clinical parameters, with most variations resulting in only modest changes in incremental costs per couple. The analysis indicates that uncertainty is driven primarily by parameters governing the effectiveness of the ICSI pathway, particularly the probability of pregnancy following ICSI. Under pessimistic assumptions regarding ICSI pregnancy success, the SFT pathway becomes cost-increasing, reflecting inefficient allocation of couples to a higher-cost treatment pathway that requires additional cycles without achieving pregnancy. We also note a 1.7% false positive rate in the test which may impact overall projected cost savings [20]. Consequently, a small proportion of couples may be referred to ICSI without the actual necessity of the treatment.

Expanding our perspective to a global perspective, over one million IVF cycles were performed worldwide in 2021 [51, 52]. In principle, our illustrative model has shown that implementing an effective SFT could reduce cost and conserve valuable resources globally, particularly in areas with limited access to fertility services. More importantly, it could spare countless couples from the devastating heartbreak of failed cycles, offering hope where uncertainty once stood.

### Policy and commissioning implications

Any SFT would be evaluated as a predictive biomarker from a NICE health technology assessment perspective [53]. In addition to direct health benefits, the framework would also consider diagnostic accuracy of the test, downstream impact on treatment allocation and cost consequences [53]. However, the absence of quality-adjusted life years (QALYs) and incremental cost-effectiveness ratios (ICERs) limits direct comparison with NICE cost-effectiveness thresholds. Based on current data, these measures are infeasible given the lack of validated evidence linking SFT to cumulative live birth or long-term health-related quality of life. Arguably, it is also challenging to measure the direct cost-effectiveness of infertility treatment as it involves the creation of life rather than improving or extending it, a view supported by Keller and Chambers [54]. Importantly, prior to clinical implementation, practical aspects to consider would include establishing a nationally agreed NHS tariffs for SFT and clarity regarding laboratory infrastructure and workforce requirements. Prospective validation studies must also be performed to demonstrate reproducible diagnostic performance across centres.

Despite current constraints, the SFT pathway could be considered under a cost-minimisation or service efficiency framework, particularly where the intervention demonstrably reduces avoidable failed IVF cycles and improves pathway efficiency without increasing overall expenditure.

### Limitations

While our study presents potential cost benefits of implementing a SFT within the NHS, several limitations must be acknowledged. Firstly, our analysis relies on data from current healthcare systems, meaning that our estimated cost savings are based on conservative assumptions. Although a potential SFT is available, it has not been adopted into the healthcare system. Without real-world data from an implemented SFT, our projections remain theoretical, albeit based on existing economic and clinical trends.

The magnitude of cost savings and improvements in patient care will depend significantly on the test’s reliability in identifying sperm dysfunction and the cost of performing the test. As SFT technology advances and validation studies are conducted, the decision-analytic model will require refinement to reflect real-world outcomes. In particular, future models should account for cumulative live birth rates and the contribution of frozen embryo transfer cycles. These factors were not included in the present analysis due to the current lack of robust evidence linking SFT results to these downstream outcomes and their inclusion would require assumptions that are highly speculative which would compromise the reliability of our findings. Additionally, our study assumes that integrating an SFT into clinical pathways will lead to reduced rates of fertilisation failure by enabling more personalised treatment strategies. However, the actual impact on fertilisation success rates and subsequent cost savings will require validation through clinical trials and post-implementation analysis. As further evidence emerges, refinements in clinical pathways and economic analyses is essential to fully realise the benefits of this novel diagnostic tool.

Lastly, we acknowledge that the economic performance of any SFT is inherently influenced by its positive and negative predictive values, which depend on both test accuracy and the underlying prevalence of the particular sperm dysfunction. In populations with lower prevalence, a greater proportion of positive results would represent false positives, potentially leading to unnecessary use of ICSI and reduced cost savings. In contrast, populations with a higher prevalence of sperm dysfunction would improve the PPV and strengthen the economic case for testing. Therefore, it is important that we consider careful patient selection and local prevalence estimates prior to implementation of the test.

## Conclusion

Our study highlights the potential of integrating SFT into clinical practice, moving beyond the current trial-and-error approach in fertility care. This advancement could reduce unnecessary IVF cycles, leading to significant cost savings, minimising emotional distress, and ultimately improving reproductive outcomes for our patients.

Given the current workload pressures and financial constraints on healthcare systems worldwide, there is an urgent need for investment in fertility diagnostics, especially with regards to male reproductive health which remains disproportionately overlooked, placing the burden and risks of treatment solely on female partners. The cost benefit analysis presented in our study also highlighted both the economic feasibility of incorporating of an SFT and also its transformative potential in delivering equitable and efficient fertility care. We anticipate that the introduction of an SFT or a multi-test approach with high predictive capabilities could lead to cumulative cost benefits and enable more personalised treatment.

We take this opportunity to implore policymakers, research institutions, and industrial representatives to prioritise the development and implementation of validated SFT. Notably, the CatFlux assay (SFT) modelled in this paper is not ready for implementation in clinical practice until comprehensive clinical and economic validation studies are concluded. The bench-to-bedside process can only be achieved through adequate funding, regulatory support, and interdisciplinary collaboration. By having a reliable SFT, we can offer couples struggling with unexplained infertility a clearer path forward, one defined by scientific precision, personalised care and renewed hope.

## Supplementary Material

Supplementary Material 1.

## Data Availability

The code used for the cost-benefit analysis in the UK and its results are detailed in Appendix A and are executed in the Interactive Python Notebook available at this link: https://colab.research.google.com/drive/1RuDg6CjKOf_P_SqH8vnYhJsfFLxcNeqQ?usp. An interactive chart showcasing the output using different predictive values can be viewed on this Google Sheets document: ⁠ https://docs.google.com/spreadsheets/d/16T2-DsUKAwpkyOeGjZ6JC3KxkElcL7TtcMsxagXx52Y/.

## Abbreviations

CART: Classification and regression tree
ICSI: Intracytoplasmic sperm injection
IVF: In vitro fertilisation
MAR: Medically assisted reproduction
NPV: Negative predictive value
OWSA: One–way sensitivity analysis
PPV: Positive predictive value
SFT: Sperm function test(s)
TFF: Total fertilisation failure
