# Supplementary material for: Cost-benefit potential of sperm function testing to the treatment pathway for unexplained infertility: a modelling study from the UK NHS perspective
Source: Health Econ Rev. 2026 Mar 6;16:45. doi: 10.1186/s13561-026-00749-5 (PMC13077886; doi:10.1186/s13561-026-00749-5)
Supplement: Supplementary file 1 — Supplementary Material 1. [file 13561_2026_749_MOESM1_ESM.docx]

**Appendix A – Coding**

**Interactive Python Notebook:** https://colab.research.google.com/drive/1RuDg6CjKOf_P_SqH8vnYhJsfFLxcNeqQ?usp

**Interactive Chart:**

https://docs.google.com/spreadsheets/d/16T2-DsUKAwpkyOeGjZ6JC3KxkElcL7TtcMsxagXx52Y/

**The Decision Tree Class & Utility Functions**

# Decision Tree Class

class DecisionTree:

def __init__(self, name, probability, value=None):

self.name = name

self.probability = probability

self.value = value or 0

self.children = []

def create_tree(node_data):

node = DecisionTree(node_data['name'], node_data['probability'], node_data.get('value'))

for child_data in node_data.get('children', []):

child = create_tree(child_data)

node.children.append(child)

return node

# Utility Functions

def calculate_expected_value(node, cumulative_probability=1.0, accumulated_value=0):

# Update the accumulated value with the value of the current node

current_accumulated_value = accumulated_value + node.value

if node.children:

return sum(calculate_expected_value(child, cumulative_probability * node.probability, current_accumulated_value) for child in node.children)

else:

node.expected_cost = cumulative_probability * node.probability * current_accumulated_value

return node.expected_cost

def add_nodes_edges(graph, node, parent=None, is_end_node=False):

node_name = node.name

if is_end_node:

label_text = f'{node_name}\n(Value: {node.value}, Expected cost: {node.expected_cost:.2f})'

else:

label_text = f'{node_name}\n(Value: {node.value})'

# Wrap text to avoid it spilling out.

node_label = textwrap.fill(label_text, width=20) # You can adjust the width as needed

graph.add_node(node_name, label=node_label)

if parent:

graph.add_edge(parent, node_name, label=f'{node.probability}')

for child in node.children:

add_nodes_edges(graph, child, parent=node_name, is_end_node=not bool(child.children))

def draw_tree(root, name='Decision Tree'):

tree_graph = nx.DiGraph()

add_nodes_edges(tree_graph, root, is_end_node=not bool(root.children))

pos = nx.drawing.nx_agraph.graphviz_layout(tree_graph, prog='dot', args='-Grankdir=LR')

node_labels = nx.get_node_attributes(tree_graph, 'label')

edge_labels = nx.get_edge_attributes(tree_graph, 'label')

plt.figure(figsize=(18, 12))

nx.draw(tree_graph, pos, labels=node_labels, with_labels=True, node_size=7000, node_color='skyblue', font_size=10, node_shape='s', arrows=True)

nx.draw_networkx_edge_labels(tree_graph, pos, edge_labels=edge_labels, font_color='red', font_size=10)

plt.title(name)

plt.show()

**Define the Decision Trees**

def get_icsi_tree():

return {

"name": "First ICSI",

"probability": 1.0,

"value": 6000,

"children": [

{

"name": "Successful First ICSI",

"probability": 0.95,

"value": 0,

"children": [

{

"name": "Successful First ICSI Pregnancy",

"probability": 0.43,

"value": 0,

"children": []

},

{

"name": "Failed First ICSI Pregnancy (Second ICSI)",

"probability": 0.57,

"value": 6000,

"children": [

{

"name": "Successful Second ICSI Pregnancy (1)",

"probability": 0.43,

"value": 0,

"children": []

},

{

"name": "Failed Second ICSI Pregnancy (Third ICSI) (1)",

"probability": 0.57,

"value": 6000,

"children": []

}

]

}

]

},

{

"name": "Failed First ICSI (Second ICSI)",

"probability": 0.05,

"value": 6000,

"children": [

{

"name": "Successful Second ICSI",

"probability": 0.60,

"value": 0,

"children": [

{

"name": "Successful Second ICSI Pregnancy (2)",

"probability": 0.43,

"value": 0,

"children": []

},

{

"name": "Failed Second ICSI Pregnancy (Third ICSI) (2)",

"probability": 0.57,

"value": 6000,

"children": []

}

]

},

{

"name": "Failed Second ICSI (Third ICSI)",

"probability": 0.40,

"value": 6000,

"children": []

}

]

}

]

}

def get_ivf_tree():

return {

"name": "First IVF",

"probability": 1.0,

"value": 5000,

"children": [

{

"name": "Successful First IVF",

"probability": 0.9,

"value": 0,

"children": [

{

"name": "Successful First IVF Pregnancy",

"probability": 0.45,

"value": 0,

"children": []

},

{

"name": "Failed First IVF Pregnancy (Second IVF)",

"probability": 0.55,

"value": 5000,

"children": [

{

"name": "Successful Second IVF Pregnancy",

"probability": 0.45,

"value": 0,

"children": []

},

{

"name": "Failed Second IVF Pregnancy (Third IVF)",

"probability": 0.55,

"value": 5000,

"children": []

}

]

}

]

},

{

"name": "Failed First IVF (First ICSI)",

"probability": 0.1,

"value": 6000,

"children": [

{

"name": "Successful First ICSI",

"probability": 0.95,

"value": 0,

"children": [

{

"name": "Successful First ICSI Pregnancy",

"probability": 0.43,

"value": 0,

"children": []

},

{

"name": "Failed First ICSI Pregnancy (Second ICSI)",

"probability": 0.57,

"value": 6000,

"children": []

}

]

},

{

"name": "Failed First ICSI (Second ICSI)",

"probability": 0.05,

"value": 6000,

"children": []

}

]

}

]

}

**Calculate the average cost per patient (current pathway)**

root = create_tree(get_ivf_tree())

expected_value_control = calculate_expected_value(root)

print(f"Expected cost (£) current pathway: {expected_value_control:.2f}")

**Expected Savings for different sensitivities (0-10%) of SFT**

# create a list of probabilities from 0.01 to 1.0 in steps of 0.001

probabilities = [i / 1000 for i in range(0, 100)]

# for each probability, calculate the expected value for each tree

expected_values = []

for probability in probabilities:

# read the IVF tree

tree_data_ivf = get_ivf_tree()

# find the child of the root node where name is "Successful First IVF" and update the probability

tree_data_ivf["children"][0]["probability"] += probability

tree_data_ivf["children"][1]["probability"] -= probability

assert tree_data_ivf["children"][0]["name"] == "Successful First IVF"

assert (

tree_data_ivf["children"][0]["probability"]

+ tree_data_ivf["children"][1]["probability"]

== 1.0

)

# calculate the expected value for the normal tree

root_ivf = create_tree(tree_data_ivf)

expected_value_ivf = calculate_expected_value(root_ivf)

# calculate the expected value for the alternate tree

tree_data_icsi = get_icsi_tree()

root_icsi = create_tree(tree_data_icsi)

expected_value_icsi = calculate_expected_value(root_icsi)

# calculate the expected value for the combined tree

expected_value_combined = (

expected_value_ivf * (1 - probability) + expected_value_icsi * probability

)

# calculate the expected values plus a fixed cost of £50

expected_value_combined_plus_sft = expected_value_combined + 50

expected_values.append(

{

"probability": probability,

"expected_value": expected_value_combined,

"expected_value_plus_sft": expected_value_combined_plus_sft,

"expected_value_normal": expected_value_ivf,

"expected_value_alternate": expected_value_icsi,

"expected_value_ivf_p": expected_value_ivf * (1 - probability),

"expected_value_icsi_p": expected_value_icsi * probability,

}

)

**Plot Probability & Savings Chart**

Interest:

A - Sensitivity of SFT (p)
B - Expected Cost of Current Clinical Pathway
C - Expected Cost of IVF-first route [ X1 ]
D - Expected Cost of IVF-first route within SFT pathway [ X1(1-p) ]
E - Expected Cost of ICSI-first route [ X2 ]
F - Expected Cost of ICSI-first route within SFT pathway [ X2(p) ]
G - Expected Cost of Sperm Function Test (SFT) Pathway [ E(X) ]
H - Expected Savings of Sperm Function Test (SFT) Pathway

We can put the entire table in the appendix.
For the main text, we’ll use columns A, B, D, F, G, H

# read expected_values into a pandas dataframe

df = pd.DataFrame(expected_values)

# Add a column for savings

df["savings"] = expected_value_control - df["expected_value_plus_sft"]

# Create the figure and a left axis for the expected_value

fig, ax1 = plt.subplots(figsize=(10, 6))

# Plot the expected value vs probability

sns.lineplot(

data=df,

x="probability",

y="expected_value_plus_sft",

ax=ax1,

label="Expected Cost of CatFlux Test Treatment Pathway Per Patient",

legend=False,

)

# Get lines and labels from ax1

lines, labels = ax1.get_legend_handles_labels()

# Create a right axis for savings using the same x-axis

ax2 = ax1.twinx()

# Set the limits for the right y-axis, which is for savings

ax2.set_ylim(1.1 * df["savings"].min(), 1.1 * df["savings"].max())

# Plot the savings vs probability with savings on the secondary y-axis

sns.lineplot(

data=df,

x="probability",

y="savings",

ax=ax2,

color="r",

label="Expected Savings of CatFlux Test Treatment Pathway Per Patient",

legend=False,

)

# Add savings line and label to existing legend info

lines2, labels2 = ax2.get_legend_handles_labels()

lines += lines2

labels += labels2

# Set labels for axes

ax1.set_xlabel("Sensitivity of CatFlux Test")

ax1.set_ylabel("Expected Cost of CatFlux Test Treatment Pathway Per Patient (£)")

ax2.set_ylabel("Expected Savings of CatFlux Test Treatment Pathway Per Patient (£)")

# Set plot title

plt.title(

"Expected Cost of CatFlux Test Treatment Pathway Per Patient vs Sensitivity of CatFlux Test"

)

# Plot the control expected value line

ax1.axhline(y=expected_value_control, color="r", linestyle="--")

control_label = f"Expected Cost of Current Clinical Pathway Per Patient: £{expected_value_control:.2f}"

lines.append(plt.Line2D([0], [0], color="r", linestyle="--"))

labels.append(control_label)

# Plot a verticle line at 2.3 probability

plt.axvline(x=0.023, color="g", linestyle="--")

# control label

control_label = f"Suggested CatSper abnormality = 1.2% - 2.3%"

lines.append(plt.Line2D([0], [0], color="g", linestyle="--"))

labels.append(control_label)

# find the intersection point of savings at 2.3%

savings_at_2_3 = df[df["probability"] == 0.023]["savings"].values[0]

# Draw a crosshair marker at the intersection point

ax2.plot(

0.023, savings_at_2_3, "yo", markersize=10

) # 'yo' is for yellow circle ('y') with fill ('o')

# Plot a vertical line at 1.2%

plt.axvline(x=0.012, color="g", linestyle="--")

# find the intersection point of savings at 1.2%

savings_at_1_2 = df[df["probability"] == 0.012]["savings"].values[0]

# draw a crosshair marker at the intersection point

ax2.plot(

0.012, savings_at_1_2, "yo", markersize=10

) # 'yo' is for yellow circle ('y') with fill ('o')

# plot a label with a formatted text box at the intersection point for 2.3%

bbox_props = dict(boxstyle="round,pad=0.3", fc="green", ec="black", lw=1, alpha=0.3)

ax2.text(

0.0245,

(savings_at_2_3 - 12),

f"expected savings per patient at 2.3%: £{savings_at_2_3:.2f}",

ha="left",

va="bottom",

fontsize=9,

bbox=bbox_props,

fontweight="bold",

)

# plot a label with a formatted text box at the intersection point for 1.2%

bbox_props = dict(boxstyle="round,pad=0.3", fc="black", ec="black", lw=1, alpha=0.3)

ax2.text(

0.0135,

savings_at_1_2 - 12,

f"expected savings per patient at 1.2%: £{savings_at_1_2:.2f}",

ha="left",

va="bottom",

fontsize=9,

bbox=bbox_props,

fontweight="bold",

)

# now the intersection point of expected value at 2.3%

expected_value_at_2_3 = df[df["probability"] == 0.023][

"expected_value_plus_sft"

].values[0]

# now the intersection point of expected value at 1.2%

expected_value_at_1_2 = df[df["probability"] == 0.012][

"expected_value_plus_sft"

].values[0]

# Draw a crosshair marker at the intersection point

ax1.plot(

0.023, expected_value_at_2_3, "yo", markersize=10

) # 'yo' is for yellow circle ('y') with fill ('o')

# Draw a crosshair marker at the intersection point

ax1.plot(

0.012, expected_value_at_1_2, "yo", markersize=10

) # 'yo' is for yellow circle ('y') with fill ('o')

# plot a label with a formatted text box at the intersection point for 2.3%

bbox_props = dict(boxstyle="round,pad=0.3", fc="red", ec="black", lw=1, alpha=0.3)

ax1.text(

0.0245,

(expected_value_at_2_3 + 5),

f"expected cost per patient at 2.3%: £{expected_value_at_2_3:.2f}",

ha="left",

va="bottom",

fontsize=9,

bbox=bbox_props,

fontweight="bold",

)

# plot a label with a formatted text box at the intersection point for 1.2%

bbox_props = dict(boxstyle="round,pad=0.3", fc="red", ec="black", lw=1, alpha=0.3)

ax1.text(

0.0135,

expected_value_at_1_2 + 5,

f"expected cost per patient at 1.2%: £{expected_value_at_1_2:.2f}",

ha="left",

va="bottom",

fontsize=9,

bbox=bbox_props,

fontweight="bold",

)

# Create the legend outside to the right

legend = ax1.legend(

lines,

labels,

loc="lower center", # The corner of the legend box...

bbox_to_anchor=(0.5,-0.35), # ...placed at these coordinates (x, y)

# borderaxespad=2,

)

# Crucial: Adjust the layout so the legend isn't cut off

plt.show()


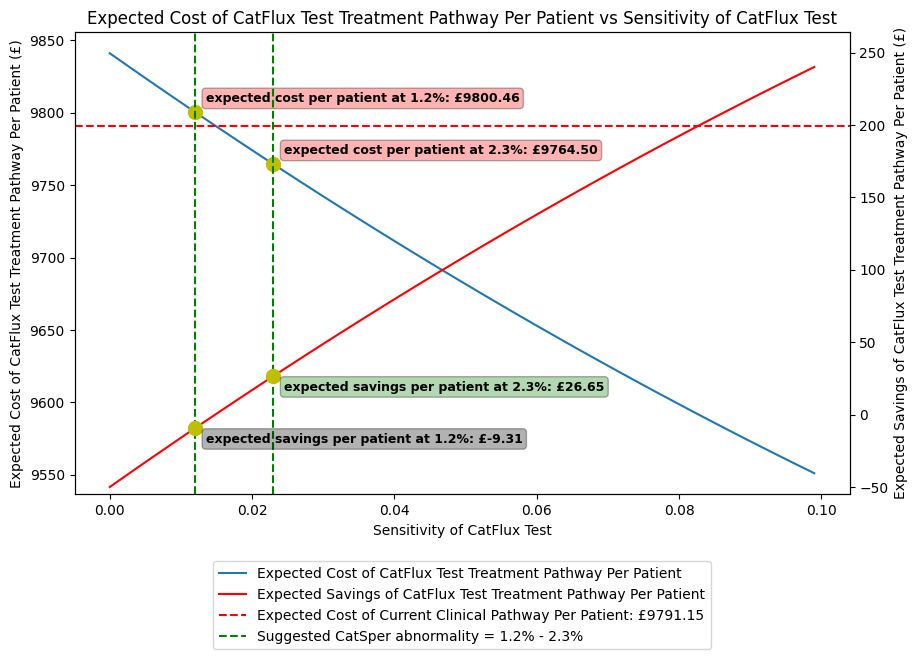


**Display Dataframe**

df["expected_value_ivf"] = df["expected_value_normal"][0]

# rename the columns

# A - Sensitivity of SFT (p)

# B - Expected Cost of Current Clinical Pathway

# C - Expected Cost of IVF-first route [ X1 ]

# D - Expected Cost of IVF-first route within SFT pathway [ X1(1-p) ]

# E - Expected Cost of ICSI-first route [ X2 ]

# F - Expected Cost of ICSI-first route within SFT pathway [ X2(p) ]

# G - Expected Cost of Sperm Function Test (SFT) Pathway [ E(X) ]

# H - Expected Savings of Sperm Function Test (SFT) Pathway

df.columns = [

"Sensitivity of Spermatozoa Function Test, p",

"Expected Cost of Sperm Function Test (SFT) Pathway, E(X)",

"Expected Cost of Sperm Function Test (SFT) Pathway, E(X) Plus CatFLux Test Cost",

"Expected Cost of IVF-first route, X1",

"Expected Cost of ICSI-first route, X2",

"Expected Cost of IVF-first route within SFT pathway, X1(1-p)",

"Expected Cost of ICSI-first route within SFT pathway, X2(p)",

"Expected Savings of Sperm Function Test (SFT) Pathway",

"Expected Cost of Current Clinical Pathway",

]

# put all to 2 decimal places except for the Sensitivity of Spermatozoa Function Test, p column

df["Sensitivity of Spermatozoa Function Test, p"] = df["Sensitivity of Spermatozoa Function Test, p"].map(

"{:.3f}".format

)

df = df.round(2)

display(df)

|  | **Sensitivity of Spermatozoa Function Test, p** | **Expected Cost of Sperm Function Test (SFT) Pathway, E(X)** | **Expected Cost of Sperm Function Test (SFT) Pathway, E(X) Plus CatFLux Test Cost** | **Expected Cost of IVF-first route, X1** | **Expected Cost of ICSI-first route, X2** | **Expected Cost of IVF-first route within SFT pathway, X1(1-p)** | **Expected Cost of ICSI-first route within SFT pathway, X2(p)** | **Expected Savings of Sperm Function Test (SFT) Pathway** | **Expected Cost of Current Clinical Pathway** |
| --- | --- | --- | --- | --- | --- | --- | --- | --- | --- |
| **0** | 0.000 | 9791.15 | 9841.15 | 9791.15 | 11623.53 | 9791.15 | 0.00 | -50.00 | 9791.15 |
| **1** | 0.001 | 9787.70 | 9837.70 | 9785.86 | 11623.53 | 9776.08 | 11.62 | -46.55 | 9791.15 |
| **2** | 0.002 | 9784.26 | 9834.26 | 9780.58 | 11623.53 | 9761.02 | 23.25 | -43.11 | 9791.15 |
| **3** | 0.003 | 9780.84 | 9830.84 | 9775.29 | 11623.53 | 9745.96 | 34.87 | -39.69 | 9791.15 |
| **4** | 0.004 | 9777.42 | 9827.42 | 9770.00 | 11623.53 | 9730.92 | 46.49 | -36.27 | 9791.15 |
| **5** | 0.005 | 9774.01 | 9824.01 | 9764.72 | 11623.53 | 9715.89 | 58.12 | -32.86 | 9791.15 |
| **6** | 0.006 | 9770.62 | 9820.62 | 9759.43 | 11623.53 | 9700.87 | 69.74 | -29.47 | 9791.15 |
| **7** | 0.007 | 9767.23 | 9817.23 | 9754.14 | 11623.53 | 9685.87 | 81.36 | -26.08 | 9791.15 |
| **8** | 0.008 | 9763.86 | 9813.86 | 9748.86 | 11623.53 | 9670.87 | 92.99 | -22.71 | 9791.15 |
| **9** | 0.009 | 9760.49 | 9810.49 | 9743.57 | 11623.53 | 9655.88 | 104.61 | -19.34 | 9791.15 |
| **10** | 0.010 | 9757.14 | 9807.14 | 9738.29 | 11623.53 | 9640.90 | 116.24 | -15.99 | 9791.15 |
| **11** | 0.011 | 9753.79 | 9803.79 | 9733.00 | 11623.53 | 9625.94 | 127.86 | -12.64 | 9791.15 |
| **12** | 0.012 | 9750.46 | 9800.46 | 9727.71 | 11623.53 | 9610.98 | 139.48 | -9.31 | 9791.15 |
| **13** | 0.013 | 9747.14 | 9797.14 | 9722.43 | 11623.53 | 9596.03 | 151.11 | -5.99 | 9791.15 |
| **14** | 0.014 | 9743.83 | 9793.83 | 9717.14 | 11623.53 | 9581.10 | 162.73 | -2.68 | 9791.15 |
| **15** | 0.015 | 9740.53 | 9790.53 | 9711.85 | 11623.53 | 9566.17 | 174.35 | 0.62 | 9791.15 |
| **16** | 0.016 | 9737.24 | 9787.24 | 9706.57 | 11623.53 | 9551.26 | 185.98 | 3.91 | 9791.15 |
| **17** | 0.017 | 9733.96 | 9783.96 | 9701.28 | 11623.53 | 9536.36 | 197.60 | 7.19 | 9791.15 |
| **18** | 0.018 | 9730.69 | 9780.69 | 9695.99 | 11623.53 | 9521.47 | 209.22 | 10.46 | 9791.15 |
| **19** | 0.019 | 9727.43 | 9777.43 | 9690.71 | 11623.53 | 9506.58 | 220.85 | 13.72 | 9791.15 |
| **20** | 0.020 | 9724.18 | 9774.18 | 9685.42 | 11623.53 | 9491.71 | 232.47 | 16.97 | 9791.15 |
| **21** | 0.021 | 9720.94 | 9770.94 | 9680.13 | 11623.53 | 9476.85 | 244.09 | 20.21 | 9791.15 |
| **22** | 0.022 | 9717.72 | 9767.72 | 9674.85 | 11623.53 | 9462.00 | 255.72 | 23.43 | 9791.15 |
| **23** | 0.023 | 9714.50 | 9764.50 | 9669.56 | 11623.53 | 9447.16 | 267.34 | 26.65 | 9791.15 |
| **24** | 0.024 | 9711.30 | 9761.30 | 9664.27 | 11623.53 | 9432.33 | 278.96 | 29.85 | 9791.15 |
| **25** | 0.025 | 9708.10 | 9758.10 | 9658.99 | 11623.53 | 9417.51 | 290.59 | 33.05 | 9791.15 |
| **26** | 0.026 | 9704.92 | 9754.92 | 9653.70 | 11623.53 | 9402.70 | 302.21 | 36.23 | 9791.15 |
| **27** | 0.027 | 9701.74 | 9751.74 | 9648.41 | 11623.53 | 9387.91 | 313.84 | 39.41 | 9791.15 |
| **28** | 0.028 | 9698.58 | 9748.58 | 9643.13 | 11623.53 | 9373.12 | 325.46 | 42.57 | 9791.15 |
| **29** | 0.029 | 9695.43 | 9745.43 | 9637.84 | 11623.53 | 9358.34 | 337.08 | 45.72 | 9791.15 |
| **30** | 0.030 | 9692.28 | 9742.28 | 9632.56 | 11623.53 | 9343.58 | 348.71 | 48.87 | 9791.15 |
| **31** | 0.031 | 9689.15 | 9739.15 | 9627.27 | 11623.53 | 9328.82 | 360.33 | 52.00 | 9791.15 |
| **32** | 0.032 | 9686.03 | 9736.03 | 9621.98 | 11623.53 | 9314.08 | 371.95 | 55.12 | 9791.15 |
| **33** | 0.033 | 9682.92 | 9732.92 | 9616.70 | 11623.53 | 9299.34 | 383.58 | 58.23 | 9791.15 |
| **34** | 0.034 | 9679.82 | 9729.82 | 9611.41 | 11623.53 | 9284.62 | 395.20 | 61.33 | 9791.15 |
| **35** | 0.035 | 9676.73 | 9726.73 | 9606.12 | 11623.53 | 9269.91 | 406.82 | 64.42 | 9791.15 |
| **36** | 0.036 | 9673.65 | 9723.65 | 9600.84 | 11623.53 | 9255.21 | 418.45 | 67.50 | 9791.15 |
| **37** | 0.037 | 9670.58 | 9720.58 | 9595.55 | 11623.53 | 9240.51 | 430.07 | 70.57 | 9791.15 |
| **38** | 0.038 | 9667.53 | 9717.53 | 9590.26 | 11623.53 | 9225.83 | 441.69 | 73.62 | 9791.15 |
| **39** | 0.039 | 9664.48 | 9714.48 | 9584.98 | 11623.53 | 9211.16 | 453.32 | 76.67 | 9791.15 |
| **40** | 0.040 | 9661.44 | 9711.44 | 9579.69 | 11623.53 | 9196.50 | 464.94 | 79.71 | 9791.15 |
| **41** | 0.041 | 9658.42 | 9708.42 | 9574.40 | 11623.53 | 9181.85 | 476.56 | 82.73 | 9791.15 |
| **42** | 0.042 | 9655.40 | 9705.40 | 9569.12 | 11623.53 | 9167.21 | 488.19 | 85.75 | 9791.15 |
| **43** | 0.043 | 9652.40 | 9702.40 | 9563.83 | 11623.53 | 9152.59 | 499.81 | 88.75 | 9791.15 |
| **44** | 0.044 | 9649.40 | 9699.40 | 9558.54 | 11623.53 | 9137.97 | 511.44 | 91.75 | 9791.15 |
| **45** | 0.045 | 9646.42 | 9696.42 | 9553.26 | 11623.53 | 9123.36 | 523.06 | 94.73 | 9791.15 |
| **46** | 0.046 | 9643.45 | 9693.45 | 9547.97 | 11623.53 | 9108.76 | 534.68 | 97.70 | 9791.15 |
| **47** | 0.047 | 9640.48 | 9690.48 | 9542.68 | 11623.53 | 9094.18 | 546.31 | 100.67 | 9791.15 |
| **48** | 0.048 | 9637.53 | 9687.53 | 9537.40 | 11623.53 | 9079.60 | 557.93 | 103.62 | 9791.15 |
| **49** | 0.049 | 9634.59 | 9684.59 | 9532.11 | 11623.53 | 9065.04 | 569.55 | 106.56 | 9791.15 |
| **50** | 0.050 | 9631.66 | 9681.66 | 9526.83 | 11623.53 | 9050.48 | 581.18 | 109.49 | 9791.15 |
| **51** | 0.051 | 9628.74 | 9678.74 | 9521.54 | 11623.53 | 9035.94 | 592.80 | 112.41 | 9791.15 |
| **52** | 0.052 | 9625.83 | 9675.83 | 9516.25 | 11623.53 | 9021.41 | 604.42 | 115.32 | 9791.15 |
| **53** | 0.053 | 9622.93 | 9672.93 | 9510.97 | 11623.53 | 9006.88 | 616.05 | 118.22 | 9791.15 |
| **54** | 0.054 | 9620.04 | 9670.04 | 9505.68 | 11623.53 | 8992.37 | 627.67 | 121.11 | 9791.15 |
| **55** | 0.055 | 9617.17 | 9667.17 | 9500.39 | 11623.53 | 8977.87 | 639.29 | 123.98 | 9791.15 |
| **56** | 0.056 | 9614.30 | 9664.30 | 9495.11 | 11623.53 | 8963.38 | 650.92 | 126.85 | 9791.15 |
| **57** | 0.057 | 9611.44 | 9661.44 | 9489.82 | 11623.53 | 8948.90 | 662.54 | 129.71 | 9791.15 |
| **58** | 0.058 | 9608.59 | 9658.59 | 9484.53 | 11623.53 | 8934.43 | 674.16 | 132.56 | 9791.15 |
| **59** | 0.059 | 9605.76 | 9655.76 | 9479.25 | 11623.53 | 8919.97 | 685.79 | 135.39 | 9791.15 |
| **60** | 0.060 | 9602.93 | 9652.93 | 9473.96 | 11623.53 | 8905.52 | 697.41 | 138.22 | 9791.15 |
| **61** | 0.061 | 9600.12 | 9650.12 | 9468.67 | 11623.53 | 8891.08 | 709.04 | 141.03 | 9791.15 |
| **62** | 0.062 | 9597.32 | 9647.32 | 9463.39 | 11623.53 | 8876.66 | 720.66 | 143.83 | 9791.15 |
| **63** | 0.063 | 9594.52 | 9644.52 | 9458.10 | 11623.53 | 8862.24 | 732.28 | 146.63 | 9791.15 |
| **64** | 0.064 | 9591.74 | 9641.74 | 9452.81 | 11623.53 | 8847.83 | 743.91 | 149.41 | 9791.15 |
| **65** | 0.065 | 9588.97 | 9638.97 | 9447.53 | 11623.53 | 8833.44 | 755.53 | 152.18 | 9791.15 |
| **66** | 0.066 | 9586.21 | 9636.21 | 9442.24 | 11623.53 | 8819.05 | 767.15 | 154.94 | 9791.15 |
| **67** | 0.067 | 9583.46 | 9633.46 | 9436.95 | 11623.53 | 8804.68 | 778.78 | 157.69 | 9791.15 |
| **68** | 0.068 | 9580.71 | 9630.71 | 9431.67 | 11623.53 | 8790.31 | 790.40 | 160.44 | 9791.15 |
| **69** | 0.069 | 9577.98 | 9627.98 | 9426.38 | 11623.53 | 8775.96 | 802.02 | 163.17 | 9791.15 |
| **70** | 0.070 | 9575.27 | 9625.27 | 9421.09 | 11623.53 | 8761.62 | 813.65 | 165.88 | 9791.15 |
| **71** | 0.071 | 9572.56 | 9622.56 | 9415.81 | 11623.53 | 8747.29 | 825.27 | 168.59 | 9791.15 |
| **72** | 0.072 | 9569.86 | 9619.86 | 9410.52 | 11623.53 | 8732.96 | 836.89 | 171.29 | 9791.15 |
| **73** | 0.073 | 9567.17 | 9617.17 | 9405.24 | 11623.53 | 8718.65 | 848.52 | 173.98 | 9791.15 |
| **74** | 0.074 | 9564.49 | 9614.49 | 9399.95 | 11623.53 | 8704.35 | 860.14 | 176.66 | 9791.15 |
| **75** | 0.075 | 9561.83 | 9611.83 | 9394.66 | 11623.53 | 8690.06 | 871.76 | 179.32 | 9791.15 |
| **76** | 0.076 | 9559.17 | 9609.17 | 9389.38 | 11623.53 | 8675.78 | 883.39 | 181.98 | 9791.15 |
| **77** | 0.077 | 9556.53 | 9606.53 | 9384.09 | 11623.53 | 8661.51 | 895.01 | 184.62 | 9791.15 |
| **78** | 0.078 | 9553.89 | 9603.89 | 9378.80 | 11623.53 | 8647.26 | 906.64 | 187.26 | 9791.15 |
| **79** | 0.079 | 9551.27 | 9601.27 | 9373.52 | 11623.53 | 8633.01 | 918.26 | 189.88 | 9791.15 |
| **80** | 0.080 | 9548.65 | 9598.65 | 9368.23 | 11623.53 | 8618.77 | 929.88 | 192.50 | 9791.15 |
| **81** | 0.081 | 9546.05 | 9596.05 | 9362.94 | 11623.53 | 8604.55 | 941.51 | 195.10 | 9791.15 |
| **82** | 0.082 | 9543.46 | 9593.46 | 9357.66 | 11623.53 | 8590.33 | 953.13 | 197.69 | 9791.15 |
| **83** | 0.083 | 9540.88 | 9590.88 | 9352.37 | 11623.53 | 8576.12 | 964.75 | 200.27 | 9791.15 |
| **84** | 0.084 | 9538.31 | 9588.31 | 9347.08 | 11623.53 | 8561.93 | 976.38 | 202.84 | 9791.15 |
| **85** | 0.085 | 9535.74 | 9585.74 | 9341.80 | 11623.53 | 8547.74 | 988.00 | 205.41 | 9791.15 |
| **86** | 0.086 | 9533.19 | 9583.19 | 9336.51 | 11623.53 | 8533.57 | 999.62 | 207.96 | 9791.15 |
| **87** | 0.087 | 9530.66 | 9580.66 | 9331.22 | 11623.53 | 8519.41 | 1011.25 | 210.49 | 9791.15 |
| **88** | 0.088 | 9528.13 | 9578.13 | 9325.94 | 11623.53 | 8505.26 | 1022.87 | 213.02 | 9791.15 |
| **89** | 0.089 | 9525.61 | 9575.61 | 9320.65 | 11623.53 | 8491.11 | 1034.49 | 215.54 | 9791.15 |
| **90** | 0.090 | 9523.10 | 9573.10 | 9315.36 | 11623.53 | 8476.98 | 1046.12 | 218.05 | 9791.15 |
| **91** | 0.091 | 9520.60 | 9570.60 | 9310.08 | 11623.53 | 8462.86 | 1057.74 | 220.55 | 9791.15 |
| **92** | 0.092 | 9518.12 | 9568.12 | 9304.79 | 11623.53 | 8448.75 | 1069.36 | 223.03 | 9791.15 |
| **93** | 0.093 | 9515.64 | 9565.64 | 9299.51 | 11623.53 | 8434.65 | 1080.99 | 225.51 | 9791.15 |
| **94** | 0.094 | 9513.17 | 9563.17 | 9294.22 | 11623.53 | 8420.56 | 1092.61 | 227.98 | 9791.15 |
| **95** | 0.095 | 9510.72 | 9560.72 | 9288.93 | 11623.53 | 8406.48 | 1104.24 | 230.43 | 9791.15 |
| **96** | 0.096 | 9508.27 | 9558.27 | 9283.65 | 11623.53 | 8392.42 | 1115.86 | 232.88 | 9791.15 |
| **97** | 0.097 | 9505.84 | 9555.84 | 9278.36 | 11623.53 | 8378.36 | 1127.48 | 235.31 | 9791.15 |
| **98** | 0.098 | 9503.42 | 9553.42 | 9273.07 | 11623.53 | 8364.31 | 1139.11 | 237.73 | 9791.15 |
| **99** | 0.099 | 9501.01 | 9551.01 | 9267.79 | 11623.53 | 8350.28 | 1150.73 | 240.14 | 9791.15 |

**Expected Savings with changes in the Probability Tree**

change_probability_tree_dict = [

{

"test_name": "current clinical pathway",

"kv_change_dict": [],

},

{

"test_name": "decreasing IVF cost to £4500 from £5000",

"kv_change_dict": [

{

"tree_name": "IVF",

"key_to_change": "First IVF",

"type": "value",

"new_value": 4500,

},

{

"tree_name": "IVF",

"key_to_change": "Failed First IVF Pregnancy (Second IVF)",

"type": "value",

"new_value": 4500,

},

{

"tree_name": "IVF",

"key_to_change": "Failed Second IVF Pregnancy (Third IVF)",

"type": "value",

"new_value": 4500,

},

],

"test_criteria": "IVF Cost",

"test_original_value": 5000,

"test_new_value": 4500,

"group": "IVF Cost",

},

{

"test_name": "increasing IVF cost to £6000 from £5000",

"kv_change_dict": [

{

"tree_name": "IVF",

"key_to_change": "First IVF",

"type": "value",

"new_value": 6000,

},

{

"tree_name": "IVF",

"key_to_change": "Failed First IVF Pregnancy (Second IVF)",

"type": "value",

"new_value": 6000,

},

{

"tree_name": "IVF",

"key_to_change": "Failed Second IVF Pregnancy (Third IVF)",

"type": "value",

"new_value": 6000,

},

],

"test_criteria": "IVF Cost",

"test_original_value": 5000,

"test_new_value": 6000,

"group": "IVF Cost",

},

{

"test_name": "decreasing IVF fertilisation failure to 5% from 10%",

"kv_change_dict": [

{

"tree_name": "IVF",

"key_to_change": "Successful First IVF",

"type": "probability",

"new_value": 0.95,

},

{

"tree_name": "IVF",

"key_to_change": "Failed First IVF (First ICSI)",

"type": "probability",

"new_value": 0.05,

},

],

"test_criteria": "IVF Fertilisation Failure",

"test_original_value": 0.1,

"test_new_value": 0.05,

},

{

"test_name": "decreasing IVF pregnancy success rate to 23% from 45%",

"kv_change_dict": [

{

"tree_name": "IVF",

"key_to_change": "Successful First IVF Pregnancy",

"type": "probability",

"new_value": 0.23,

},

{

"tree_name": "IVF",

"key_to_change": "Failed First IVF Pregnancy (Second IVF)",

"type": "probability",

"new_value": 0.77,

},

{

"tree_name": "IVF",

"key_to_change": "Successful Second IVF Pregnancy",

"type": "probability",

"new_value": 0.23,

},

{

"tree_name": "IVF",

"key_to_change": "Failed Second IVF Pregnancy (Third IVF)",

"type": "probability",

"new_value": 0.77,

},

],

"test_criteria": "IVF Pregnancy Success",

"test_original_value": 0.45,

"test_new_value": 0.23,

},

{

"test_name": "decreasing ICSI cost to £5500 from £6000",

"kv_change_dict": [

{

"tree_name": "ICSI",

"key_to_change": "First ICSI",

"type": "value",

"new_value": 5500,

},

{

"tree_name": "ICSI",

"key_to_change": "Failed First ICSI Pregnancy (Second ICSI)",

"type": "value",

"new_value": 5500,

},

{

"tree_name": "ICSI",

"key_to_change": "Failed Second ICSI Pregnancy (Third ICSI) (1)",

"type": "value",

"new_value": 5500,

},

{

"tree_name": "ICSI",

"key_to_change": "Failed First ICSI (Second ICSI)",

"type": "value",

"new_value": 5500,

},

{

"tree_name": "ICSI",

"key_to_change": "Failed Second ICSI Pregnancy (Third ICSI) (2)",

"type": "value",

"new_value": 5500,

},

{

"tree_name": "ICSI",

"key_to_change": "Failed Second ICSI (Third ICSI)",

"type": "value",

"new_value": 5500,

},

{

"tree_name": "IVF",

"key_to_change": "Failed First IVF (First ICSI)",

"type": "value",

"new_value": 5500,

},

{

"tree_name": "IVF",

"key_to_change": "Failed First ICSI Pregnancy (Second ICSI)",

"type": "value",

"new_value": 5500,

},

{

"tree_name": "IVF",

"key_to_change": "Failed First ICSI (Second ICSI)",

"type": "value",

"new_value": 5500,

},

],

"test_criteria": "ICSI Cost",

"test_original_value": 6000,

"test_new_value": 5500,

"group": "ICSI Cost",

},

{

"test_name": "increasing ICSI cost to £7000 from £6000",

"kv_change_dict": [

{

"tree_name": "ICSI",

"key_to_change": "First ICSI",

"type": "value",

"new_value": 7000,

},

{

"tree_name": "ICSI",

"key_to_change": "Failed First ICSI Pregnancy (Second ICSI)",

"type": "value",

"new_value": 7000,

},

{

"tree_name": "ICSI",

"key_to_change": "Failed Second ICSI Pregnancy (Third ICSI) (1)",

"type": "value",

"new_value": 7000,

},

{

"tree_name": "ICSI",

"key_to_change": "Failed First ICSI (Second ICSI)",

"type": "value",

"new_value": 7000,

},

{

"tree_name": "ICSI",

"key_to_change": "Failed Second ICSI Pregnancy (Third ICSI) (2)",

"type": "value",

"new_value": 7000,

},

{

"tree_name": "ICSI",

"key_to_change": "Failed Second ICSI (Third ICSI)",

"type": "value",

"new_value": 7000,

},

{

"tree_name": "IVF",

"key_to_change": "Failed First IVF (First ICSI)",

"type": "value",

"new_value": 7000,

},

{

"tree_name": "IVF",

"key_to_change": "Failed First ICSI Pregnancy (Second ICSI)",

"type": "value",

"new_value": 7000,

},

{

"tree_name": "IVF",

"key_to_change": "Failed First ICSI (Second ICSI)",

"type": "value",

"new_value": 7000,

},

],

"test_criteria": "ICSI Cost",

"test_original_value": 6000,

"test_new_value": 7000,

"group": "ICSI Cost",

},

{

"test_name": "decreasing ICSI fertilisation failure to 1% from 5%",

"kv_change_dict": [

{

"tree_name": "ICSI",

"key_to_change": "Successful First ICSI",

"type": "probability",

"new_value": 0.99,

},

{

"tree_name": "ICSI",

"key_to_change": "Failed First ICSI (Second ICSI)",

"type": "probability",

"new_value": 0.01,

},

{

"tree_name": "IVF",

"key_to_change": "Successful First ICSI",

"type": "probability",

"new_value": 0.99,

},

{

"tree_name": "IVF",

"key_to_change": "Failed First ICSI (Second ICSI)",

"type": "probability",

"new_value": 0.01,

},

],

"test_criteria": "ICSI Fertilisation Failure",

"test_original_value": 0.05,

"test_new_value": 0.01,

},

{

"test_name": "decreasing ICSI recurrent fertilisation failure to 10% from 40%",

"kv_change_dict": [

{

"tree_name": "ICSI",

"key_to_change": "Successful Second ICSI",

"type": "probability",

"new_value": 0.9,

},

{

"tree_name": "ICSI",

"key_to_change": "Failed Second ICSI (Third ICSI)",

"type": "probability",

"new_value": 0.1,

},

],

"test_criteria": "ICSI Recurrent Fertilisation Failure",

"test_original_value": 0.4,

"test_new_value": 0.1,

},

{

"test_name": "decreasing ICSI pregnancy success rate to 16% from 43%",

"kv_change_dict": [

{

"tree_name": "ICSI",

"key_to_change": "Successful First ICSI Pregnancy",

"type": "probability",

"new_value": 0.16,

},

{

"tree_name": "ICSI",

"key_to_change": "Failed First ICSI Pregnancy (Second ICSI)",

"type": "probability",

"new_value": 0.84,

},

{

"tree_name": "ICSI",

"key_to_change": "Successful Second ICSI Pregnancy (1)",

"type": "probability",

"new_value": 0.16,

},

{

"tree_name": "ICSI",

"key_to_change": "Failed Second ICSI Pregnancy (Third ICSI) (1)",

"type": "probability",

"new_value": 0.84,

},

{

"tree_name": "ICSI",

"key_to_change": "Successful Second ICSI Pregnancy (2)",

"type": "probability",

"new_value": 0.16,

},

{

"tree_name": "ICSI",

"key_to_change": "Failed Second ICSI Pregnancy (Third ICSI) (2)",

"type": "probability",

"new_value": 0.84,

},

{

"tree_name": "IVF",

"key_to_change": "Successful First ICSI Pregnancy",

"type": "probability",

"new_value": 0.16,

},

{

"tree_name": "IVF",

"key_to_change": "Failed First ICSI Pregnancy (Second ICSI)",

"type": "probability",

"new_value": 0.84,

},

],

"test_criteria": "ICSI Pregnancy Success",

"test_original_value": 0.43,

"test_new_value": 0.16,

},

]

def change_tree(node, kv_change_dict):

# Check current node

if node["name"] == kv_change_dict["key_to_change"]:

if kv_change_dict["type"] == "probability":

node["probability"] = kv_change_dict["new_value"]

elif kv_change_dict["type"] == "value":

node["value"] = kv_change_dict["new_value"]

# Recursively search children

for child in node.get("children", []):

change_tree(child, kv_change_dict)

return node

"""

function to calculated the expected savings at 1.2% and 2.3% after replacing certain probabilities/costs with alternative values

Args:

probability_change_dict (dict): a dictionary of probabilities to change

cost_change_dict (dict): a dictionary of costs to change

Returns:

float: the expected savings at 1.2% and 2.3% for each of the scenarios

"""

def calculate_expected_savings(

change_probability_tree_dict

):

oneway_analysis_dict = []

try:

for change_probability_tree in change_probability_tree_dict:

ivf_tree = get_ivf_tree()

icsi_tree = get_icsi_tree()

for kv_change_dict in change_probability_tree["kv_change_dict"]:

if kv_change_dict["tree_name"] == "IVF":

ivf_tree = change_tree(ivf_tree, kv_change_dict)

elif kv_change_dict["tree_name"] == "ICSI":

icsi_tree = change_tree(icsi_tree, kv_change_dict)

# recalculate the expected value for the control tree

root_ivf = create_tree(ivf_tree)

expected_value_control = calculate_expected_value(root_ivf)

test_result = {

"test_name": change_probability_tree["test_name"],

"expected_savings_at_1_2_percent": None,

"expected_savings_at_2_3_percent": None,

"group": change_probability_tree["group"] if "group" in change_probability_tree else None,

"test_criteria": change_probability_tree["test_criteria"] if "test_criteria" in change_probability_tree else None,

"test_original_value": change_probability_tree["test_original_value"] if "test_original_value" in change_probability_tree else None,

"test_new_value": change_probability_tree["test_new_value"] if "test_new_value" in change_probability_tree else None,

}

original_ivf_tree_children_probability = ivf_tree["children"][0]["probability"]

original_ivf_tree_children_probability_2 = ivf_tree["children"][1]["probability"]

# repeat the cost calculation method for sft test 0.012 and 0.023

for p in [0.012, 0.023]:

ivf_tree["children"][0]["probability"] = original_ivf_tree_children_probability + p

ivf_tree["children"][1]["probability"] = original_ivf_tree_children_probability_2 - p

# calculate the expected value for the normal tree

root_ivf = create_tree(ivf_tree)

expected_value_ivf = calculate_expected_value(root_ivf)

# calculate the expected value for the alternate tree

root_icsi = create_tree(icsi_tree)

expected_value_icsi = calculate_expected_value(root_icsi)

# calculate the expected value for the combined tree

expected_value_combined = (

expected_value_ivf * (1 - p) + expected_value_icsi * p

)

expected_value_combined_plus_sft = expected_value_combined + 50

expected_savings = expected_value_control - expected_value_combined_plus_sft

if p == 0.012:

test_result["expected_savings_at_1_2_percent"] = round(

expected_savings, 2

)

elif p == 0.023:

test_result["expected_savings_at_2_3_percent"] = round(

expected_savings, 2

)

oneway_analysis_dict.append(test_result)

# break

except Exception as e:

print(e)

# print the full error

print(change_probability_tree["test_name"])

return None

return oneway_analysis_dict

**Tornado Plot using 1-way sensitivity analysis result**

# Extract control values (current clinical pathway)

control = expected_savings_1_way_sensitivity_analysis_dict[0]

control_1_2 = control["expected_savings_at_1_2_percent"]

control_2_3 = control["expected_savings_at_2_3_percent"]

# Group data by group field

grouped_data = {}

for item in expected_savings_1_way_sensitivity_analysis_dict[1:]: # Skip control

group_key = item["group"] if item["group"] else item["test_criteria"]

if group_key not in grouped_data:

grouped_data[group_key] = []

grouped_data[group_key].append(

{

"test_name": item["test_name"],

"test_criteria": item["test_criteria"],

"savings_1_2": item["expected_savings_at_1_2_percent"],

"savings_2_3": item["expected_savings_at_2_3_percent"],

"original_value": item["test_original_value"],

"new_value": item["test_new_value"],

"deviation_1_2": item["expected_savings_at_1_2_percent"] - control_1_2,

"deviation_2_3": item["expected_savings_at_2_3_percent"] - control_2_3,

}

)

# Create figure with two subplots

fig, (ax1, ax2) = plt.subplots(1, 2, figsize=(14, 8))

# Function to create tornado plot with actual savings

def create_tornado_plot_actual_savings(ax, rate_key, control_value, title_suffix):

"""Create a tornado plot showing actual savings values."""

# Prepare data for this rate

categories = []

min_values = []

max_values = []

bar_labels = []

is_range_bar = []

for group_name, group_items in grouped_data.items():

# Get savings for this rate

if rate_key == "1_2":

savings = [item["savings_1_2"] for item in group_items]

else:

savings = [item["savings_2_3"] for item in group_items]

# For single-item groups

if len(group_items) == 1:

item = group_items[0]

savings_value = savings[0]

# Show the actual savings value

min_values.append(min(savings_value, control_value))

max_values.append(max(savings_value, control_value))

is_range_bar.append(False)

# Create label with change information

if item["original_value"] is not None and item["new_value"] is not None:

if isinstance(item["original_value"], float):

label = f"{group_name}\n({item['original_value']*100:.0f}% → {item['new_value']*100:.0f}%)"

else:

label = f"{group_name}\n(£{item['original_value']} → £{item['new_value']})"

else:

label = f"{group_name}"

bar_labels.append(label)

# For multi-item groups (like cost), show both variations

else:

min_savings = min(savings)

max_savings = max(savings)

# Include control value in range if it falls outside

actual_min = min(min_savings, max_savings, control_value)

actual_max = max(min_savings, max_savings, control_value)

min_values.append(actual_min)

max_values.append(actual_max)

is_range_bar.append(True)

# Create label with change information for both items

changes = []

for item in group_items:

if item["original_value"] is not None and item["new_value"] is not None:

if isinstance(item["original_value"], float):

changes.append(f"{item['new_value']*100:.0f}%")

else:

changes.append(f"£{item['new_value']}")

if changes:

label = f"{group_name}\n({', '.join(changes)})"

else:

label = f"{group_name}"

bar_labels.append(label)

# Sort by range width (largest at top)

ranges = [abs(h - l) for l, h in zip(min_values, max_values)]

indices = np.argsort(ranges)[::-1] # Descending order

# Reorder all lists

categories = [bar_labels[i] for i in indices]

min_values = [min_values[i] for i in indices]

max_values = [max_values[i] for i in indices]

is_range_bar = [is_range_bar[i] for i in indices]

# Create horizontal bar positions

y_pos = np.arange(len(categories))

# Plot bars - color based on whether value is above/below control

for i, (y, min_val, max_val) in enumerate(zip(y_pos, min_values, max_values)):

# Split bar at control value for visual clarity

if min_val < control_value and max_val > control_value:

# Bar crosses control - split into two colors

ax.barh(

y,

control_value - min_val,

left=min_val,

color="lightcoral",

edgecolor="darkred",

height=0.7,

)

ax.barh(

y,

max_val - control_value,

left=control_value,

color="lightgreen",

edgecolor="darkgreen",

height=0.7,

)

elif max_val <= control_value:

# Entire bar is below or at control

ax.barh(

y,

max_val - min_val,

left=min_val,

color="lightcoral",

edgecolor="darkred",

height=0.7,

)

else:

# Entire bar is above control

ax.barh(

y,

max_val - min_val,

left=min_val,

color="lightgreen",

edgecolor="darkgreen",

height=0.7,

)

# Add control line

ax.axvline(

x=control_value,

color="black",

linewidth=2,

linestyle="-",

alpha=0.8,

label=f"Control: £{control_value:.1f}",

)

# add zero line (---)

ax.axvline(

x=0,

color="grey",

linewidth=1,

linestyle="--",

alpha=0.8,

)

rate_title = "1.2%" if rate_key == "1_2" else "2.3%"

# Set labels and title

ax.set_yticks(y_pos)

ax.set_yticklabels(categories, fontsize=10)

# ax.set_xlabel("Expected Savings (£)", fontsize=11, fontweight="bold")

ax.set_xlabel(

f"Deviation of expected savings from base-case value\nat {rate_title} CatFlux predictive rate (£)",

fontsize=11,

fontweight="bold",

)

# ax.set_title(

# f"Expected Savings at {rate_title} Predictive Rate\n{title_suffix}",

# fontsize=13,

# fontweight="bold",

# pad=15,

# )

# Add grid

ax.grid(True, axis="x", alpha=0.3, linestyle="--")

# Add legend

ax.legend(loc="upper right", fontsize=10)

# Set x-axis limits with some padding

all_values = min_values + max_values + [control_value]

x_min = min(all_values)

x_max = max(all_values)

padding = (x_max - x_min) * 0.1 # 10% padding

ax.set_xlim(x_min - padding, x_max + padding)

# Add value labels on bars at ends

for i, (min_val, max_val) in enumerate(zip(min_values, max_values)):

# Label at minimum end if it's not zero-width bar

if abs(max_val - min_val) > 0.01:

# Format with 1 decimal place

min_text = f"{min_val:.1f}"

max_text = f"{max_val:.1f}"

# Position labels slightly inside the bar ends

offset = (x_max - x_min) * 0.01

# Label at minimum end

ax.text(

min_val - offset,

i,

min_text,

ha="right",

va="center",

fontsize=9,

fontweight="bold",

color="darkred" if min_val < control_value else "darkgreen",

bbox=dict(facecolor="white", alpha=0.7, edgecolor="none", pad=1),

)

# Label at maximum end

ax.text(

max_val + offset,

i,

max_text,

ha="left",

va="center",

fontsize=9,

fontweight="bold",

color="darkred" if max_val < control_value else "darkgreen",

bbox=dict(facecolor="white", alpha=0.7, edgecolor="none", pad=1),

)

# Create tornado plot for 1.2% rate

# create_tornado_plot_actual_savings(ax1, "1_2", control_1_2, "Actual Savings Values")

create_tornado_plot_actual_savings(

ax1, "1_2", control_1_2, "compared to current clinical pathway"

)

# Create tornado plot for 2.3% rate

# create_tornado_plot_actual_savings(ax2, "2_3", control_2_3, "Actual Savings Values")

create_tornado_plot_actual_savings(

ax2, "2_3", control_2_3, "compared to current clinical pathway"

)

# Adjust layout

plt.tight_layout()

# plt.suptitle('Tornado Plot: Sensitivity Analysis of IVF/ICSI Parameters\n(Showing Actual Expected Savings)',

plt.suptitle(

"One-way sensitivity analysis of IVF and ICSI Parameters",

fontsize=16,

fontweight="bold",

y=1.02,

)

plt.show()

# Create a summary table

print("\n" + "=" * 100)

print("SUMMARY: Expected Savings and Deviations from Control")

print("=" * 100)

print(

f"{'Parameter':<40} {'Variation':<25} {'1.2% Savings (£)':<15} {'2.3% Savings (£)':<15} {'Dev 1.2%':<12} {'Dev 2.3%':<12}"

)

print("-" * 100)

for group_name, group_items in grouped_data.items():

for item in group_items:

# Extract the variation direction

test_name = item["test_name"]

if "decreasing" in test_name:

direction = "↓"

elif "increasing" in test_name:

direction = "↑"

else:

direction = ""

# Format the variation

if item["original_value"] is not None and item["new_value"] is not None:

if isinstance(item["original_value"], float):

variation = f"{direction} {item['original_value']*100:.0f}% → {item['new_value']*100:.0f}%"

else:

variation = (

f"{direction} £{item['original_value']} → £{item['new_value']}"

)

else:

variation = ""

# Get parameter name (shortened)

param_name = item["test_criteria"]

if len(param_name) > 38:

param_name = param_name[:35] + "..."

print(

f"{param_name:<40} {variation:<25} {item['savings_1_2']:>10.2f}{'':<4} {item['savings_2_3']:>10.2f}{'':<4} {item['deviation_1_2']:>8.2f}{'':<3} {item['deviation_2_3']:>8.2f}"

)

print("=" * 100)

print(f"\nCONTROL (Current Clinical Pathway):")

print(f" • Expected savings at 1.2% rate: £{control_1_2:.2f}")

print(f" • Expected savings at 2.3% rate: £{control_2_3:.2f}")


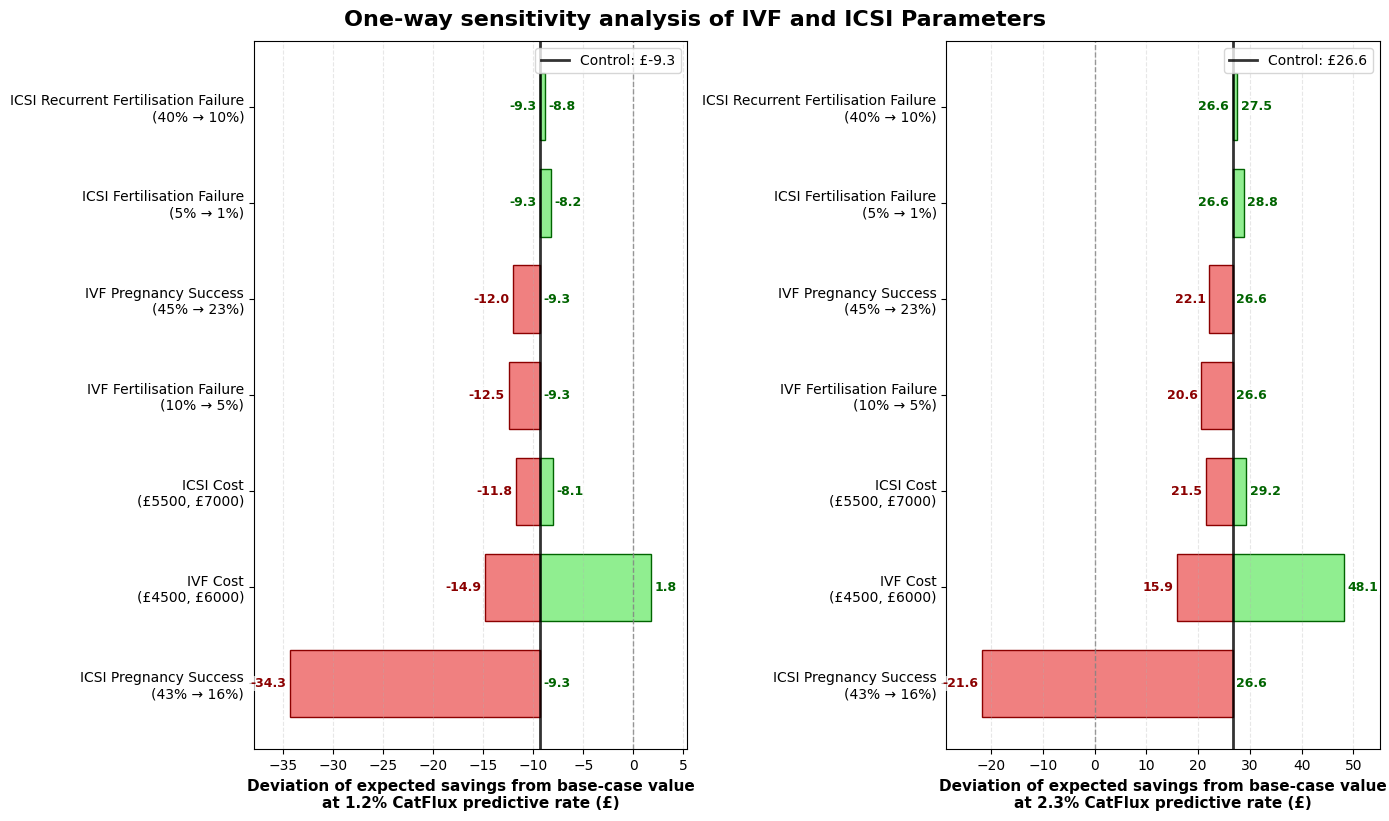


SUMMARY: Expected Savings and Deviations from Control

====================================================================================================

Parameter Variation 1.2% Savings (£) 2.3% Savings (£) Dev 1.2% Dev 2.3%

----------------------------------------------------------------------------------------------------

IVF Cost ↓ £5000 → £4500 -14.86 15.90 -5.55 -10.75

IVF Cost ↑ £5000 → £6000 1.79 48.14 11.10 21.49

IVF Fertilisation Failure ↓ 10% → 5% -12.48 20.57 -3.17 -6.08

IVF Pregnancy Success ↓ 45% → 23% -12.01 22.13 -2.70 -4.52

ICSI Cost ↓ £6000 → £5500 -8.08 29.22 1.23 2.57

ICSI Cost ↑ £6000 → £7000 -11.78 21.51 -2.47 -5.14

ICSI Fertilisation Failure ↓ 5% → 1% -8.22 28.77 1.09 2.12

ICSI Recurrent Fertilisation Failure ↓ 40% → 10% -8.85 27.54 0.46 0.89

ICSI Pregnancy Success ↓ 43% → 16% -34.31 -21.65 -25.00 -48.30

====================================================================================================

CONTROL (Current Clinical Pathway):

• Expected savings at 1.2% rate: £-9.31

• Expected savings at 2.3% rate: £26.65
